# Reconciliation between operational taxonomic units and species boundaries

**DOI:** 10.1093/femsec/fix029

**Published:** 2017-03-21

**Authors:** Mohamed Mysara, Peter Vandamme, Ruben Props, Frederiek-Maarten Kerckhof, Natalie Leys, Nico Boon, Jeroen Raes, Pieter Monsieurs

**Affiliations:** 1 Unit of Microbiology, Belgian Nuclear Research Centre (SCK-CEN), 2400 Mol, Belgium; 2 Department of Bio-Engineering sciences, Vrije Universiteit Brussel (VUB), 1050 Brussels, Belgium; 3 VIB lab for Bioinformatics and (eco-)systems biology, VIB, 3000 Leuven, Belgium; 4 Department of Microbiology and Immunology, REGA institute, KU Leuven, 3000 Leuven, Belgium; 5 Department of Biochemistry and microbiology, Ghent University, 9000 Ghent, Belgium; 6 Department of Biochemical and microbial technology, Ghent University, 9000 Ghent, Belgium

**Keywords:** 16S rRNA amplicon sequencing, microbial biodiversity, next generation sequencing, operational taxonomic units (OTUs), OTU clustering, 16S rRNA metagenomics, DynamiC

## Abstract

The development of high-throughput sequencing technologies has revolutionised the field of microbial ecology via 16S rRNA gene amplicon sequencing approaches. Clustering those amplicon sequencing reads into operational taxonomic units (OTUs) using a fixed cut-off is a commonly used approach to estimate microbial diversity. A 97% threshold was chosen with the intended purpose that resulting OTUs could be interpreted as a proxy for bacterial species. Our results show that the robustness of such a generalised cut-off is questionable when applied to short amplicons only covering one or two variable regions of the 16S rRNA gene. It will lead to biases in diversity metrics and makes it hard to compare results obtained with amplicons derived with different primer sets. The method introduced within this work takes into account the differential evolutional rates of taxonomic lineages in order to define a dynamic and taxonomic-dependent OTU clustering cut-off score. For a taxonomic family consisting of species showing high evolutionary conservation in the amplified variable regions, the cut-off will be more stringent than 97%. By taking into consideration the amplified variable regions and the taxonomic family when defining this cut-off, such a threshold will lead to more robust results and closer correspondence between OTUs and species. This approach has been implemented in a publicly available software package called DynamiC.

## Teaser

More accurate and robust estimates of microbial diversity are generated when performing an OTU clustering step using a dynamic cut-off that integrates bacterial taxonomy and differential evolutionary rates.

## INTRODUCTION

A major breakthrough in microbial ecology has been realised by the usage of PCR-based amplification of phylogenetic marker genes, such as the 16S rRNA gene for the assessment of microbial diversity in a specific environment, thereby bypassing time-consuming and challenging isolation and cultivation approaches. The usage of this culture-independent approach has been accelerated via the introduction of high-throughput sequencing technologies, leading to a dramatic increase of marker gene sequencing studies for the assessment of microbial communities.

However, downstream processing of those large amounts of sequencing data is not standardised. In the most straightforward approach, the reads resulting from the high-throughput sequencing platforms are grouped and classified based on their sequence similarity to a reference taxonomic dataset, a methodology most often referred to as phylotyping. However, such a binning procedure will be biased towards the existing taxonomic classification—including its classification errors—which is largely based on cultivable organisms (Amann, Ludwig and Schleifer 1995; Wang *et al.*^2007^; Rosselló-Móra 2012). Therefore, the most widely used bioinformatics pipelines like mothur (Schloss *et al.*^2009^), QIIME (Caporaso *et al.*^2010^) and UPARSE (Edgar 2013) implemented an alternative approach, where all sequencing reads are grouped together based on their sequence similarity to each other rather than a reference taxonomic dataset, resulting in cluster of reads, referred to as operational taxonomic units (OTUs).

For clustering those reads into OTUs, a similarity threshold of 97% is frequently used for pragmatic reasons, as it offers a compromise between the potential inflation of the number of OTUs due to sequencing errors and the cut-off used for taxonomic classification. Indeed, on the one side, sequencing errors—either introduced by the PCR or the sequencing platform—might introduce artificial OTUs, thereby leading to an overestimation of the microbial diversity (Huse *et al.*^2007^; Quince *et al.*^2009^; Edgar 2013; Eren *et al.*2013b). On the other side, it has already been suggested that this cut-off of 97% underestimates the total microbial diversity (Pedrós-Alió 2006; Chen *et al.*^2013^; Koeppel and Wu 2013; Eren *et al.*2013a), as bacterial taxonomists nowadays use a more stringent cut-off of 98.65% to delineate species (Stackebrandt and Ebers 2006; Janda and Abbott 2007; Kim *et al.*^2014^). It should however be stressed that organisms that share more than 98.65% of their full 16S rRNA gene sequences may or may not represent the same species (Fox, Wisotzkey and Jurtshuk 1992), so the potential for underestimating bacterial diversity through 16S rRNA amplicon sequencing is intrinsic to the molecule studied and cannot be avoided. Moreover, as traditional clustering algorithms do not offer a high resolution when using a fixed global threshold, advanced algorithms have been developed resolving fine-scale variation without inflating the amount of OTUs caused by sequencing errors, e.g. DADA2 (Callahan *et al.*^2016^), Swarm (Mahé *et al.*^2014^), distribution-based clustering (Preheim *et al.*^2013^), MED (Eren *et al.*^2015^) and CROP (Hao, Jiang and Chen 2011).

Although the current definition of a bacterial species has been a matter of debate for many decades (Hutchinson 1968; Mayr 1982; Brenner, Staley and Krieg 2001; Cohan and Perry 2007; Doolittle and Zhaxybayeva 2009; Staley 2009; Connor *et al.*^2010^), today the delineation of bacterial species is not only based on the sequence similarity of (near) entire 16S rRNA genes, but also requires a certain conservation of phenotype and whole genome sequence similarity, now commonly determined through the analysis of average sequence identities of shared genes (Richter and Rosselló-Móra 2009) or genome-to-genome distance parameters (Meier-Kolthoff *et al.*^2013^). Yet, the 16S rRNA amplicon sequencing approach is exploiting a culture-independent approach, aiming at the identification of bacterial species that cannot be grown under laboratory conditions. As such, phenotypical information or whole genome sequences cannot be obtained for the majority of species within the investigated microbial communities. Therefore, researchers can only rely on the 16S rRNA gene for assigning a taxonomic class (Curtis, Sloan and Scannell 2002; Venter *et al.*^2004^; Giovannoni and Stingl 2005; Staley 2006; Koeppel and Wu 2013). Moreover, the currently most widely used sequencing platform for amplicon-based microbial community assessment—being the Illumina MiSeq platform—is only able to sequence a small subregion (nowadays mostly covering the V3–V4 regions) of the 16S rRNA gene thereby ignoring the fact that the sequence similarity threshold scores are derived for full-length sequences. Given the fact that the degree of conservation for each variable region might vary between taxonomic lineages, the region(s) chosen for amplification can have a huge impact on the microbial diversity observed (Schmalenberger, Schwieger and Tebbe 2001; Clarridge 2004; Yu and Morrison 2004). Moreover, applying a general cut-off for OTU clustering is based on the naïve assumption that 16S rRNA genes evolve at the same rate regardless of their taxonomic affiliation (Schloss and Westcott 2011; Schmidt, Matias Rodrigues and von Mering 2014; Yarza *et al.*^2014^). This might result in merging two distinct taxonomic species into one OTU when the conservation is higher than expected, or a splitting up of one species over different OTUs when more sequence variation is observed. Since the recommended amplicon targeted for 16S rRNA gene sequencing has been varying due to frequent updates of the sequencing technologies and corresponding selection of primer pairs, this will lead to biases in the assessment of microbial diversity, hampering direct comparison of diversity indices between studies targeting different regions of the 16S rRNA gene (Youssef *et al.*^2009^; Schloss and Westcott 2011).

Several efforts have been made to address the limits of one or a limited number of variable regions by quantifying the loss of information, as well as the effect of using different (combinations of) variable regions on richness and phylogeny (Chakravorty *et al.*^2007^; Liu *et al.*^2008^; Youssef *et al.*^2009^; Jeraldo, Chia and Goldenfeld 2011; Kumar *et al.*^2011^; Soergel *et al.*^2012^; Ghyselinck *et al.*^2013^; Yarza *et al.*^2014^). Performing the above-mentioned OTU clustering on sequencing data obtained for amplicons covering one or two variable regions within the 16S rRNA gene will lead to important shortcomings concerning the comparability and interpretation of bacterial diversities. Therefore, within this work, we first show based on a recent SILVA 16S rRNA gene reference database that applying a 97% cut-off during the clustering step results in a biased interpretation of the OTU clustering results, as the variability observed for a specific variable region within a taxonomic family might vary dramatically within the bacterial kingdom. Next, we propose an innovative ad hoc approach for OTU clustering using a dynamic threshold score, instead of a fixed one, adapted for each bacterial family. Such an approach would compensate for the difference in evolutionary rates within the different taxonomic lineages throughout their various variable regions, as such resulting in OTUs reflecting more accurately the species level. Finally, this family-dependent threshold approach is validated using a 16S rRNA gene sequencing dataset obtained from synthetic and real microbial communities.

## MATERIALS AND METHODS

The majority of the analyses performed within this work rely on type strains as stored in the high-quality and curated SILVA database (part of the Living Tree Project, LTP) (Yarza *et al.*^2008^). We used release 119 of this database where we grouped all species based on their taxonomic family, omitting those families containing overall less than three species or those with pending classification (see Table 1). It is important to emphasise that such a database contains for each species only the type strain represented by only a single copy of the 16S rRNA gene.

**Table 1.** Illustration the number of Bacteria and Archaea within the LTP database release 119: raw data, cleaned data (omitting sequences with unclear classification) and filtered data (omitting sequences belonging to families consisting of one or two sequences).

	Raw		Cleaned		Filtered	
Domain	Bacteria	Archaea	Bacteria	Archaea	Bacteria	Archaea
**Phyllum**	33	2	30	2	22	2
**Class**	67	9	57	9	39	8
**Order**	162	18	119	16	89	12
**Family**	327	31	280	28	216	17
**Genus**	2111	118	1937	115	1884	103
**Total**	10850	419	10511	411	10462	395

To study the level of variability within each taxonomic family, the selected 16S rRNA sequences were analysed using the SSU-ALIGN software package (version 0.1) (Nawrocki 2009) for determining the degree of evolutionary conservation for each position within the 16S rRNA gene per family, which can be quantified by assigning a conservation bit score to each position within this rRNA gene (ranging between 0 and 2, with 0 indicating a highly variable position and 2 a highly conserved position). The bit score is calculated as follows:

\begin{equation*}
{\rm{bit\_score}} = {\log _2}(4) - \left( - \frac{1}{W}\sum\limits_{l = 1}^W {\sum\limits_{b = A}^T {q_l^b} } \log_2 (q_l^b)\right)
\end{equation*}

where W is the length of the alignment and $q_l^b$ is the frequency of nucleotide *b* at position *l* in the alignment. When using this bit score in the results section to emphasise high variability of certain regions, we use two minus the bit score as parameter for easy interpretation, in the remainder of the manuscript called the bit-score complement. Nine hypervariable regions together with the intervening conserved regions were defined for all 16S rRNA sequences using the *Escherichia coli* 16S rRNA gene as reference, as described by Ashelford *et al.* (2005). Throughout the analysis described in the Results section, analyses were performed on artificial amplicons, produced using two different approaches: (1) combining one until nine adjacent hypervariable regions (e.g. for a combination of seven variable regions, the artificial amplicons produced are V1–V7, V2–V8 and V3–V9, see Fig. 2) and (2) producing sequences of 300 or 500 nucleotides using a 50-bp sliding window (e.g. position 1–300, 51–350, 101–400, etc., see Table 2). The latter approach tries to mimic the effect of working with short sequencing reads, currently producing amplicon sequences roughly varying between 300 and 500 bp.

**Table 2.** Illustration of the percentage of overmerged OTUs upon applying both the 97% cut-off and our proposed lookup table, for two amplicon lengths (300 and 500 bp) and a sliding window of 50 bp.

Position		Overmerged OTUs (%)			Position		Overmerged OTUs (%)		
Start	End	0.03 Cut-off	Lookup	ii) Lookup*	Start	End	0.03 Cut-off	Lookup	Lookup*
1	300	22	15	17	1	500	25	16	19
51	350	27	18	20	51	550	29	18	21
101	400	34	20	25	101	600	31	19	23
151	450	29	18	21	151	650	31	19	22
201	500	45	24	29	201	700	45	24	28
251	550	51	28	33	251	750	48	25	31
301	600	44	25	30	301	800	48	25	31
351	650	38	23	27	351	850	43	24	28
401	700	40	24	27	401	900	45	24	28
451	750	53	28	33	451	950	55	27	33
501	800	55	29	34	501	1000	47	25	31
551	850	48	26	32	551	1050	44	24	29
601	900	54	28	34	601	1100	49	25	30
651	950	63	32	36	651	1150	49	25	30
701	1000	48	26	32	701	1200	49	25	31
751	1050	48	26	31	751	1250	46	24	29
801	1100	47	26	30	801	1300	44	24	29
851	1150	49	26	32	851	1350	48	25	30
901	1200	48	25	32	901	1400	48	24	29
951	1250	41	24	28	951	1450	41	22	26
1001	1300	50	26	31					
1051	1350	53	28	34					
1101	1400	53	27	33					
1151	1450	49	25	30					
Average		44	24	29	Average		43	23	28

The lookup table was calculated using either the complete number of species for each family (column ‘lookup’) or subsampled families containing at maximum of seven species per family (column ‘lookup*’).

Either starting from complete 16S rRNA sequences directly obtained from the reference database or starting from partially compiled sequences of these genes (as mentioned in the paragraph above), OTUs were obtained for both approaches using the mothur (version 1.33.3) commands *align.seqs, dist.seqs* and *cluster* for alignment, distance calculation and clustering, respectively. For distance calculation, the default one-gap option in mothur was selected, implementing the method proposed by Sogin *et al.*(2006), while for clustering the average neighbourhood approach was selected, following Schloss, Gevers and Westcott (2011), using various thresholds as a cut-off.

For the analysis of the synthetic mock community, Illumina MiSeq sequencing data were obtained from the published samples of 56 species (59 strains) in 34 samples (Schirmer *et al.*^2015^) (samples were subsampled to 25 000 reads, and samples with a lower read count were removed from the data set). The data were downloaded from the European Nucleotide Archive under the accession number: PRJEB6244 (http://www.ebi.ac.uk/ena/data/view/PRJEB6244). The amplicon spans the V4 variable region or the combination V3–V4. The mock sequencing data were preprocessed using mothur, including the commands *make.contigs, align.seqs, screen.seqs, filter.seqs, dist.seqs* and *cluster*. Chimeric sequences were identified via the mothur command *seq.error*, before performing the distance calculation. The resulting OTUs were filtered by removing rare OTUs (those representing <0.1% of the number of subsampled reads).

Finally, to illustrate the real-life performance of our newly proposed methodology, we used a dataset generated in the context of the Human Microbiome Project (HMP), consisting of 16S rRNA gene amplicon sequencing data obtained for 15 to 18 body sites from 300 healthy human individuals. The mothur-treated version of this data set is available via http://www.hmpdacc.org/HMMCP/healthy/. Those samples for which sequencing data were obtained for all three different amplicons (V1–V3, V3–V5 and V6–V9) were selected. Filtering out those samples with <1000 reads for any of those amplicons led to a total of 64 samples. The number of sequences for each sample was normalised throughout the three amplicons, via randomly subsampling them to the sample with the smallest read count. These data were used to assess whether the preprocessing pipeline predicted the same absolute number of OTUs when applied on different amplicons. The correlation between the numbers of OTUs predicted for each amplicon was analysed using ordinary least squares regression. Normality of the residuals was evaluated by visual inspection of the quantile–quantile plot, and errors on the model parameters were corrected for heteroscedasticity (Breusch-Pagan test, *P* < 0.0001), using the HC0 correction available from the sandwich package (v2.3.4, vcovHC function). The quality of the regression was tested using the Wald test (*P* < 0.0001), and regression lines derived for different pipelines were compared using the glht function (Tukey's all-pair comparison) from the multcomp package (v1.4.5) as implemented in R.

Additionally, we downloaded from the Sequencing Read Archive extra data sets to test the behaviour of our newly developed approach in other environments: PRJNA347423 (V3–V4 region, soil microbiome), PRJNA182049 and PRJNA253784 (V4–V5 region, mesophilic anaerobic digestion microbiome), PRJNA316325 (V5–V6 region, rizosphere microbiome) and PRJNA302180 (V3–V4, cooling water). All samples were analysed in the same way as the MiSeq mock samples, i.e. using the mothur commands *make.contigs, align.seqs, screen.seqs, filter.seqs, pre.cluster, chimera.uchime, dist.seqs* and *cluster*.

As one of the evaluation criteria we will use the average improvement of our DynamiC clustering method compared to the default approach using a fixed 97% cut-off during OTU clustering. This percentage is defined as:

\begin{equation*}
\hspace{130pt}\frac{{\left({{\rm{number\ of\ OTUs\ using\ DynamiC}} {-} {\rm{number\ of\ OTUs\ using\ }}97{\rm{\% \ cut}} {-} {\rm{off}}} \right)}}{{{\rm{Correct\ number\ of\ species}}}}
\end{equation*}

## RESULTS

### Divergent evolution of variable regions of the 16S rRNA gene

**Figure 1.**
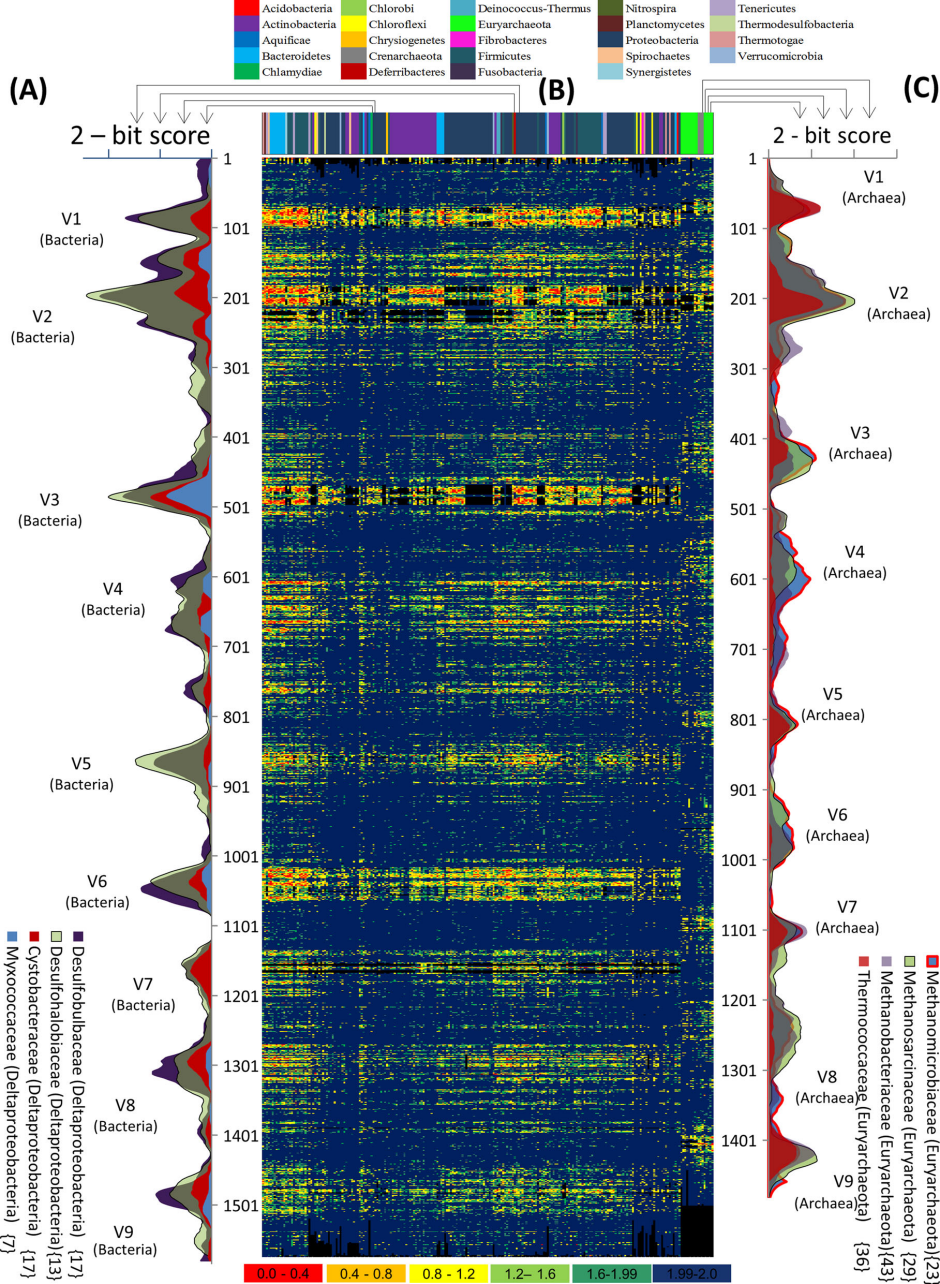
Different levels of conservation within the 16S rRNA gene compared between bacterial and archaeal families that fulfilled the selection criteria (*n* = 233). Within the heatmap, each column represents a taxonomic family, and each row represents the position within the 16S rRNA gene, starting from the 5΄ start site (top) until the 3΄ end of the gene (bottom). The colour code of the cells reflects the alignment bit score (as derived using the SSU-ALIGN algorithm), with red indicating the most variable and blue the most conserved positions as shown in section (**B**). A black colour means that this position is not covered in that taxonomic family. To emphasise the variation within taxonomically related groups, four families belonging to the same bacterial class (Deltaproteobacteria) and four belonging to the same archaeal class (Euryarchaeota) were selected and the normalised bit-score (bit score averaged over a 30-nucleotide window) was plotted against the position within the 16S rRNA gene, as shown in panels **A** and **C**.

In the most ideal scenario, each OTU would represent a taxonomic species. However, as stated above, the current OTU clustering approaches using a fixed 97% threshold will not be able to achieve this goal. To further assess to which extent the current approach is deviating from the optimal scenario, all sequences selected from the SILVA database were aligned using the SSU-align algorithm. Subsequently, for each taxonomic family separately, the conservation of each position within the 16S rRNA gene is translated into a bit score, which was plotted via a heatmap (Fig. 1 and supplementary file 1). From this heatmap, it is clear that there is a distinction in evolutionary conservation of the variable regions between different taxonomic families. As an example to emphasise these differences between the various families, we zoomed in on eight families, i.e. four families belonging to the same bacterial class (Deltaproteobacteria) and four belong to the same archaeal class (Euryarchaeota) (see Fig. 1A and C). Although the selected families contain a comparable number of species (between 7 and 17 for the selected Deltaproteobacteria families and between 23 and 43 for the selected Euryarchaeota families), each family showed a different profile when it came to the level of conservation throughout its various regions. Moreover, upon comparing two families, the pattern of their conservation throughout the various regions is not consistent, e.g. for the family Methanomicrobiaceae (23 species) and family Methanosarcinaceae (29 species)—albeit belonging to the same taxonomic class—the first family shows significantly more conservation in V9 compared to the latter family, while the opposite is true for region V4. Additional examples are shown in supplementary file 2.

Moreover, as the families do not contain the same number of species, we assessed whether the same observation could be made when comparing families containing a comparable number of species. Families were divided into three groups: families containing a low number of species (3–10 species per family), an intermediate number (11–40 species) or high number (41 or more species). Each category had a similar number of families compared to the other ones: the low, intermediate and high category consisted of 84, 72 and 77 families, respectively. Upon investigating the plots for each category, various degrees of conservations were observed within the same variable region of the different families (data not shown).

### Robustness of OTU clustering when combining different 16S rRNA hypervariable and conserved regions

**Figure 2.**
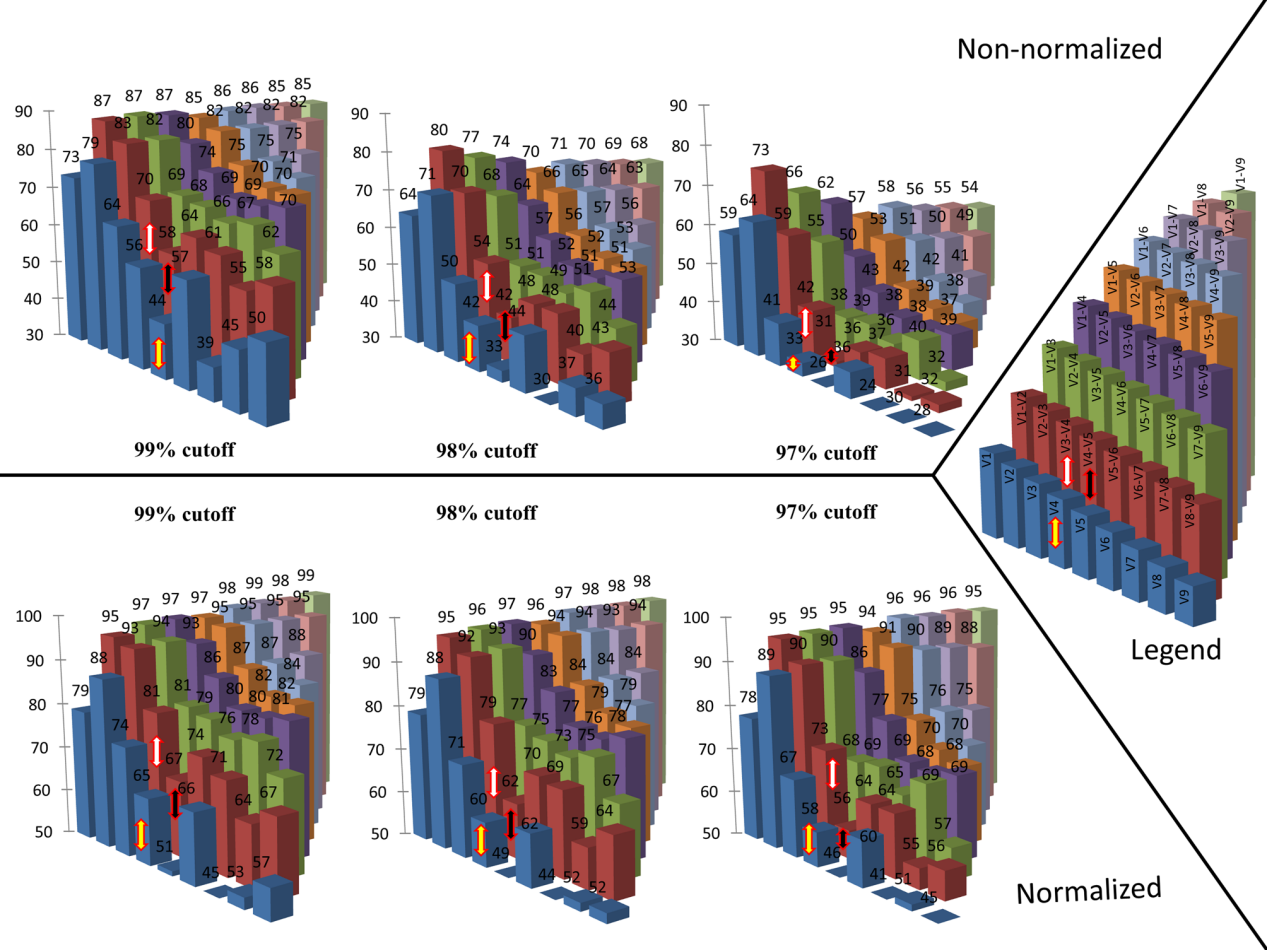
Barplot illustrating the percentage of retrieved OTUs upon applying the various cut-offs (99%, 98% and 97%), thereby using various combinations of variable regions, ranging from one region to nine variable regions (see the legend on the right). The analysis was repeated twice, the first time (upper part) performed on the total number of species within each family, while the second time (lower part) performed after cleaning up the database by only retaining one representative species per family (in case two or more had a similarity higher than the applied cuff-off). The total amount of OTUs that should be returned is 10.857 for complete database and 9.199, 8.246 and 5.822 for the curated database based on a cut-off of 99%, 98% and 97%, respectively. The currently most used amplicons V4, V3–V4 and V4–V5 are indicated with a yellow, white and black arrow, respectively.

To examine the discriminative power of the various variable regions, hypothetical amplicons with increasing lengths covering various adjacent variable regions were subsequently clustered into OTUs (see Fig. 2). The aim of this analysis is to assess the potential erroneous merging of different species within the same OTU. In the most ideal scenario, each species within this analysis would be represented by one single OTU, implying that the total number of OTUs would equal the number of tested species.

However, when applying the commonly used 97% similarity as cut-off for the OTU clustering step on the complete LTP database, depending on the combination of hypervariable regions tested, all species are clustered into a total number of OTUs between 2586 and 7948 (i.e. between 24% and 73% of the expected number of OTUs, respectively). Even increasing the OTU clustering cut-off value to 98% and 99% still uncovered a significant portion of erroneously merged species, i.e. the analysis returned between 30% and 80% of the expected number of OTUs for 98%, and between 39% and 87% of the expected numbers of OTUs for the 99% cut-off, respectively (Fig. 2).

In addition, investigating the discriminatory power of the commonly used amplicon covering the V3–V4 regions using the Illumina MiSeq platform, this 97% threshold approach was able to return 42% of expected number of OTUs. On the contrary, for the V1 and V2 regions separately, the level of overmerging—i.e. grouping two sequences from different species into one OTU, but that should end up in separate OTUs—was found very comparable to that of the whole 16S rRNA gene length upon applying the 99% cut-off. However, upon applying less stringent cut-off values (98% and 97%), the variable regions V1 and V2 resulted in a more accurate OTU clustering compared to the full length of the gene, as the latter one takes into account the less variable second half of the 16S rRNA gene compared to the first half, which is in line with previous findings (Youssef *et al.*^2009^).

Importantly, even when using the complete 16S rRNA gene, an erroneous merging of distinct species into one OTU was observed for 46% of the species upon using 97% threshold. Also for the higher cut-off score of 98%, 98.65% and 99%, discrepancies could be found between the number of OTUs and the number of species, leading to overmerging up to 32%, 21% and 15% of the species, respectively (data for cut-off score 98.65% not shown).

In order to compensate for this bias, the database was cleaned in such a way that only one representative species was retained in case a sequence similarity higher than the applied cut-off was found, as such resulting in a curated database complying with a similarity cut-off score of 97%, 98% or 99%, respectively. We repeated the analysis mentioned above on this curated database for different cut-off thresholds and different combinations of variable regions. However, even when working with this curated database, our analysis still resulted in significant erroneous merging, as between 45% and 99%, between 44% and 98% and between 41% and 96% of the expected number of OTUs was returned for the 99%, 98% and 97% cut-off, respectively. Interestingly, even when testing all the combinations containing the two highly informative regions V1 and V2, on average 5% of all species were erroneously merged with another one upon using the 97% cut-off. Similarly, if we examined the amplicons including only two variable regions, 5%–36%, 5%–41% and 5%–45% of the species are erroneously merged upon applying a 99%, 98% and 97% cut-off, respectively.

### Robustness of OTU clustering using short read lengths

Where the previous analysis allows to gain insight into the discriminative power of combining different 16S rRNA gene regions, the next analysis deals with a situation where a sequencing technology permits to sequence amplicons of a specific length (300 and 500 nucleotides in the hypothetical examples described below, as those lengths correspond to amplicon lengths currently produced by sequencing platforms). The question to be answered is which part of the 16S rRNA gene would result in the most accurate OTU clustering i.e. OTUs reflecting most accurately the bacterial taxonomic classification. Using a 50-bp sliding window, we ended up with 24 artificial amplicons with a length of 300 nucleotides and 20 amplicons with a length of 500 nucleotides. For each of those artificial amplicons, all pairwise distances between the species within one family were calculated (see Table 2).

For each of the windows, the distances obtained by pairwise alignment of all sequences were ranked per family, allowing us to identify the minimum, the lower 2.5%, the lower 10%, the lower 25%, the median, the higher 25% and the maximum distances observed between each two sequences originating from the same family. These different distance distributions demonstrate the differences between all species within a family, in a heatmap plot (see supplementary file 3). Windows including V1 and V2 showed the highest variability within each family compared to amplicons spanning the V5, V7 and V8 region. As for the window covering the nowadays commonly amplified V3 and V4 region on the Illumina MiSeq platform, the percentage of overmerged OTUs is 45% (Table 2, window 201–700). Based on this analysis, the best window to be selected for amplicon sequencing would be region starting at position 1 until 300 and position 1 until 500, provided that you have a sequencing technology allowing high-quality amplicons with lengths up to 300 bp or 500 bp, respectively.

### Dynamic family-dependent cut-off for OTU clustering

**Figure 3.**
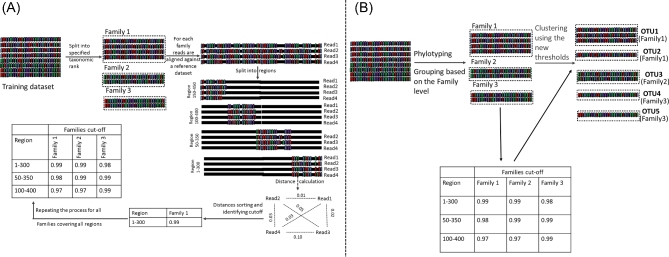
Illustration of the proposed lookup table approach for defining a dynamic threshold for OTU clustering of 16S rRNA sequencing reads, explaining the training step (**A**) and execution step (B). (**A**) In the training step, for each taxonomic family the cut-off score for a specified amplicon is calculated using a reference database, as such resulting in a lookup table reporting all cut-off scores. (**B**) This cut-off lookup table is used in the execution step for clustering sequencing reads into OTUs using a dynamic clustering implementation.

Based on the observations outlined above, as thoroughly illustrated in supplementary file 3, it is clear that defining one generally applicable cut-off for OTU clustering is very difficult, as the selected amplicon and the taxonomic family are important parameters influencing an appropriate cut-off. Therefore, we developed a new approach which dynamically defines the cut-offs applied when performing the OTU clustering step. Basically, instead of using a rigid cut-off of e.g. 97%, we propose a dynamic way for defining these cut-off scores, which will vary depending on the taxonomic family and the targeted amplicon. The approach developed within this work generally consists of two steps: a training step and an execution step. In the training step, for each taxonomic family the cut-off score for a specified amplicon is calculated using a reference database (in our case the LTP). As illustrated in Fig. 3A, these cut-off scores are calculated as follows: (1) for all species within this family—based on the LTP taxonomic database—the sequence fragment corresponding to this amplicon is derived. (2) All those fragments are aligned and the distance scores are ordered. (3) As we considered 2.5% of these distances to be outliers, the distance corresponding to the 2.5 percentile is used as OTU clustering cut-off. The 2.5% percentile was selected as using the minimum value (0th percentile) was shown to result in the separation of different strains or different copies of 16S rRNA gene of the same species into various OTUs, while a higher percentile resulted in only a marginal improvement compared to the default 97% cut-off approach (data not shown). (4) If this value is <1% or >3%, this cut-off value is set at 1% or 3%, respectively. Repeating those four steps for all taxonomic families results in a lookup table defining the OTU clustering cut-off per family.

After calculating the cut-off values in the training step, this cut-off lookup table is used in a second step for clustering sequencing reads into OTUs using a dynamic clustering implementation (Fig. 3B). Therefore, in a first step each read is assigned to a specific taxonomic family. After this, for each taxonomic family separately the OTU grouping is performed using a user-defined clustering algorithm, thereby using the cut-off value for that family as found in the previously constructed lookup table (see Fig. 3B). For reads with uncertain taxonomic classification at the family level (e.g. due to sequencing errors or due to the presence of less well-studied bacterial species), we propose using the default cut-off (by default set to 97% but this value can be adapted by the user). The software—entitled DynamiC (Dynamic Cut-off)—to create such a lookup table and apply it for the OTU clustering of any amplicon sequencing data set using a preferred taxonomic reference database is freely available via https://github.com/M-Mysara/DynamiC/. It can use any taxonomic level to calculate the clustering thresholds (Kingdom to Genus) using the training mode of the tool (using the _m option). Additionally, the default cut-off can be changed using the _Z option.

As a proof of concept, before we tested this approach on real-life sequencing data, we applied this approach to the LTP reference database. Lookup tables were produced for each of the sliding window approaches mentioned above (windows of 300 and 500 bp, using a shift of 50 bp). Using such a lookup table to create OTUs per taxonomic family, we observed that the erroneous merging of different species within the same OTU was reduced to 24% while the default clustering cut-off resulted in 44% overmerging (Table 2). However, as the average cut-off score calculated over the complete lookup table was 2.2% and 2.4% for 300 and 500 bp, respectively, we also compared our approach with a traditional OTU clustering approach using a 2% distance score as cut-off (instead of the default 3% used previously). Also in this case, we noticed an improvement (i.e. less erroneous clustering) with our method compared to the 2% rigid cut-off approach (15% of the species wrongly merged with our approach compared to 22% using the rigid approach) (data not shown). Similarly, applying a cut-off score of 98.65%—a value nowadays frequently used to distinguish different species in bacterial taxonomy (Kim *et al.*^2014^)—still results in an improvement of 21% with our method compared to the rigid cut-off approach (data not shown). Apart from this theoretical approach, the DynamiC tool was also tested on the nowadays most commonly used amplicons, i.e. V1–V2, V3–V4, V4 and V4–V5 (see supplementary file 4), thereby showing an average improvement of 7%, 25%, 22% and 28% for the V1–V2, V3–V4, V4 and V4–V5 regions, respectively. This analysis also allows us to identify those bacterial classes that would benefit the most from our approach. For example, samples dominated by bacteria belonging to the Acidobacteria class would benefit significantly from our approach, while less benefit should be expected from microbiomes dominated by *Clostridia*.

However, the question might be raised whether our lookup table method is sensitive to the size of the taxonomic family, i.e. can we also determine a reliable cut-off if the taxonomic family only contains a limited number of species, or do we only obtain reliable cut-off with sufficiently covered taxonomic families. Therefore, to mimic such a situation we randomly subsampled each family to have at most seven species and subsequently recalculated the lookup table as described above. Applying the same approach as mentioned above to the complete dataset, however with the subsampled lookup table, resulted in a similar level of improvement as previously reported i.e. a reduction to 28% overmerging using DynamiC compared to 43% using the 3% cut-off for the 500 bp region (see Table 2).

### Comparative analysis using mock data

Next, we tested our lookup table method on a mock dataset consisting of 34 samples, each of them comprising 56 species. After preprocessing, either our lookup table or the commonly used 97% cut-off was applied to cluster those reads into OTUs. However, not all 56 species could be detected in all samples, i.e. no sequencing reads were found for some species that should theoretically be present. This might be due to the fact that some of the species were added in low concentrations. Therefore, the number of detectable species—as reported in supplementary file 5—is in many cases lower than 56. In the most optimal case, the OTU clustering step should return a number of OTUs corresponding to the number of detectable species (i.e. a varying number of species, displayed in supplementary file 5) present in the mock community. From the analysis, our proposed lookup table approach lead to an average of 44.2 OTUs per sample compared to 41.6 OTUs achieved by the default 97% approach. Upon inspecting the percentage of lost species due to overmerging, our proposed approach lead to an average loss of four OTUs compared to six OTUs when applying the default cut-off of 97%. Particularly, our approach was able to successfully separate reads belonging to closely related species within the mock communities such as (1) *Chlorobium limicola, C. phaeovibrioides* and *C. phaeobacteroides*; (2) *Thermotoga neapolitana* and *T. petrophila*; and (3) *Pyrobaculum aerophilum* and *P. calidifontis*.

However, by applying a too stringent cut-off, our method might also lead to the opposite effect, thereby assigning reads originating from the same species to different OTUs (i.e. oversplitting), while those reads should be grouped in the same OTU. We noticed an oversplitting in 4% of all OTUs when applying the default 97% cut-off, while with our lookup table approach only a minor increase to 5% was observed. Most of the oversplitting for both approaches was observed within the Spirochaetaceae family and could be explained by the presence of extremely erroneous reads and the presence of multiple non-identical paralogues of the 16S rRNA genes.

### Application on real-life samples

**Figure 4.**
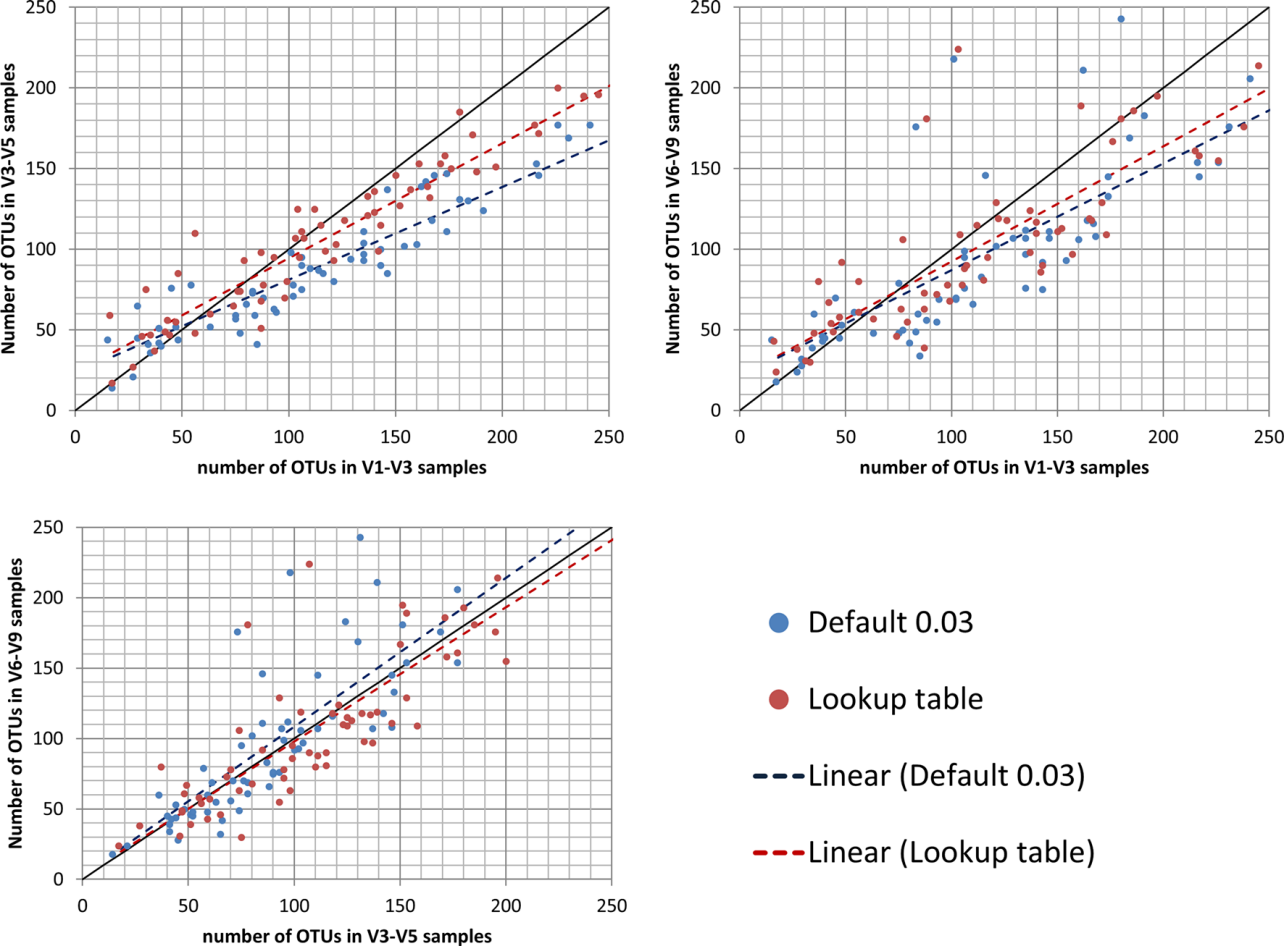
Plot illustrating the correspondence between the number of OTUs derived based on different amplicons (V1–V3, V3–V5 and V6–V9) as calculated for the HMP samples. The numbers of OTUs obtained for each amplicon were plotted against each other for all pairwise combinations. Results produced using the default approach (fixed cut-off of 3%) are coloured blue; results produced using our lookup table methodology in red. The corresponding linear fit (coloured in red or blue for both approaches, respectively) summarises the relation between the number of OTUs returned for different amplicons for both approaches; the black line represents the ideal scenario i.e. a perfect correspondence between the number of OTUs returned for both amplicons.

To illustrate the performance of our lookup table methodology compared with the default approach using a fixed threshold of 3%, we applied both approaches on microbiome samples obtained from a wide range of habitats, i.e. human gut microbiomes, soil and rizosphere microbiomes, microbiomes related to anaerobic digestion, and cooling water microbiomes. For the human gut microbiome test case, we tested both approaches on 64 samples, for which three different amplicons were sequenced (V1–V3, V3–V5 and V6–V9). Our proposed lookup table methodology will produce optimal results when the taxonomic family cannot be retrieved for only a small fraction of the reads, as was the case with this HMP data set (10%, 12% and 19% for the V1–V3, V3–V5 and V6–V9 region, respectively). As such, our method was able to produce a number of OTUs with less variability between the different amplicons i.e. the microbial diversity predicted with our methodology was more robust than the traditional approach (*P*-value < 0.01 based on a paired *t*-test comparing the standard deviations on the number of OTUs returned for the three regions between both approaches). Upon plotting numbers of OTUs obtained for each region against one of the other two regions, i.e. V1–V3 against V3–V5, V1–V3 against V6–V9 and V3–V5 against V6–V9, closer correspondence can be observed between the number of OTUs returned for the different amplicons when applying our proposed lookup table (see Fig. 4). Statistical evidence for a difference between both methods could only be obtained for V1–V3 versus V3–V5 (*P*-value < 0.0001), but not for the other two pairwise combinations (*P*-value of 0.25 and 0.10 for V1–V3 versus V6–V9 and V3–V5 versus V6–V9, respectively).

To illustrate to what extent applying our proposed approach would affect the species richness, we re-assessed the richness of the complete dataset of V3–V5 of the HMP dataset for healthy individuals (consisting of 28 million sequences from around 5000 samples) using both approaches, producing 36 000 OTUs and 29 000 respectively for the lookup table and default approach (data not shown), i.e. an increase of 24%. These results suggest that default approach might underestimate the species richness.

Finally, both approaches were tested on microbiomes derived from the other habitats. Where we reported for the human microbiome on average an increase in the number of OTUs of 24%, the microbiomes obtained from anaerobic digestion, cooling water, rhizosphere and soil samples showed an increase in the number of OTUs of 42%, 9%, 8% and 5%, respectively. The percentages of unclassified reads at the family level were 11%, 9%, 70% and 79% for anaerobic digestion, cooling water, rhizosphere and soil samples, respectively.

## DISCUSSION

In prokaryotic taxonomy, bacterial species are delineated on the basis of both genotypic and phenotypic criteria. Sequence analysis of (near) entire 16S rRNA genes cannot be used as a sole criterion to delineate species because this gene lacks resolution to distinguish between closely related species. Yet, organisms that share <98.65% of their (near) entire 16S rRNA genes consistently represent different species (Kim *et al.*^2014^). In traditional bacterial systematics, species were defined as phenotypically coherent groups of strains sharing a certain percentage of DNA–DNA hybridisation (DDH) (Vandamme *et al.*^1996^). The latter DDH experiments are being replaced by whole genome sequence-based parameters such as the degree of average nucleotide identity (ANI) of shared genes (Richter and Rosselló-Móra 2009) or the genome-to-genome distance parameter (Meier-Kolthoff *et al.*^2013^). Species delineated this way comprise phenotypically coherent strains that share a varying percentage of core genes (up to 70%) and that have a unique set of accessory genes that commonly represents up to 30% of their gene content (Lapierre and Gogarten 2009). Species defined this manner may thus group strains with an impressive functional diversity and 16S rRNA amplicon-based diversity assessments cannot take this into account. Analysis of 16S rRNA amplicon data sets should therefore avoid further underestimation of species diversity by applying rigorous analysis methods.

After high-throughput sequencing and preprocessing the obtained 16S rRNA amplicon products, sequencing reads are clustered together based on sequence similarity, a process often referred to as OTU clustering. Within this step, reads are grouped together if they have a sequence similarity higher than a predefined cut-off, for which most often a threshold of 97% is used. Subsequently, those results are used as a proxy for a taxonomic species. This fixed cut-off of 97% is selected for practical reasons, as it offers a compromise between potential inflation of the number of OTUs due to sequencing errors and a cut-off used for taxonomic classification. However, most current high-throughput sequencing technologies only allow producing short reads covering a span of one or two variable regions only. Therefore, applying such a fixed cut-off implicitly assumes that all variable regions in all taxa evolve at the same rate, an assumption which is certainly not met—as shown in previous experiments (Schmalenberger, Schwieger and Tebbe 2001; Clarridge 2004; Yu and Morrison 2004; Schloss and Westcott 2011; Schmidt, Matias Rodrigues and von Mering 2014; Yarza *et al.*^2014^) as well as the current work.

The first aim of this work was to confirm that the amount of sequence variation—and hence the evolutionary rate—was differing between different taxonomic lineages depending on the selected variable region, as already observed previously (Schmalenberger, Schwieger and Tebbe 2001; Clarridge 2004; Yu and Morrison 2004; Schloss and Westcott 2011; Schmidt, Matias Rodrigues and von Mering 2014; Yarza *et al.*^2014^). To emphasise those differences in sequence conservation throughout the 16S rRNA gene among the different taxonomic lineages, we analysed this conservation for each family separately starting from a recent release of LTP 16S rRNA gene database. The family level was used as it has been characterised as being more stable than other lower taxonomic levels (Konstantinidis and Tiedje 2005; Yarza *et al.*^2014^), yet providing a good taxonomic resolution. The level of conservation of each variable region could differ dramatically between the various families, even when belonging to the same higher taxonomic class, as shown in Fig. 1. In addition, the level of conservation is not always consistent over all the various regions within one family, which is in line with previous findings (Chakravorty *et al.*^2007^; Liu *et al.*^2008^; Jeraldo, Chia and Goldenfeld 2011; Kumar *et al.*^2011^; Soergel *et al.*^2012^). These observations have important consequences, as measurements of diversity for the same environment might lead to significantly different results when based on amplicon sequencing data derived for two amplicons covering different 16S rRNA variable regions, thereby hampering a solid comparison between studies focussing on different amplicons.

Clustering the 16S rRNA gene sequences as available in the LTP database, our results showed a clear overmerging of different taxonomic species into one OTU when using the default 97% cutoff (up to 44%, Table 2), which in practice would lead to an underestimation of the bacterial diversity. Such overmerging is patently obvious when dealing with short subregions of these 16S rRNA genes (representing different amplicons), but, interestingly, even when using the full-length sequences an overmerging of 46% of the species was observed (Fig. 2), as already shown in previous studies (Ash *et al.*^1991^; Fox, Wisotzkey and Jurtshuk 1992; Rosselló-Mora and Amann 2001; Stackebrandt and Ebers 2006; Staley 2006; Janda and Abbott 2007; Achtman and Wagner 2008; Kim *et al.*^2014^). This implies that the database contains bacteria which are assigned to different species despite the fact that they show a sequence similarity exceeding the specified cut-off (Ash *et al.*^1991^; Rosselló-Mora and Amann 2001; Stackebrandt and Ebers 2006; Kim *et al.*^2014^). This is not surprising as the classification of bacterial taxonomy is not solely based on the 16S rRNA gene sequence similarity, but also requires a minimum ANI and DDH value.

Taking together two facts mentioned above, being that (1) current high-throughput sequencing technologies only allow to sequence a subregion of the full-length 16S rRNA gene and (2) different variable regions are showing a different degree of conservation between different taxonomic families, we developed a dynamic OTU clustering approach, an ad hoc heuristic which is based upon first grouping all sequencing reads based on their taxonomic family, and next using a family-dependent cut-off for clustering the short sequencing reads into OTUs rather than using a generally applied fixed cut-off. Moreover, this dynamic clustering cut-off score is also depending on the exact subregion of the 16S rRNA gene which is amplified. Our proposed family-dependent cut-off approach was able to cluster OTUs with an increased efficiency compared to the default 97%, as erroneous merging of different species within the same OTU was reduced to 24% while the default clustering cut-off resulted in 44% overmerging (Table 2). This level of improvement was independent of the coverage of the different families (i.e. the number of species within a family). As such, the same level of efficiency can be extrapolated when dealing with novel species of existing families or with families with a high or low level of species sequenced. When applying this analysis workflow on mock samples harbouring closely related species, the approach proposed within this work allowed to estimate the richness more accurately than the default pipeline using 97% as general cut-off. Upon applying both approaches on a dataset generated in the context of the HMP, a significantly closer correspondence (reduced variability) was reported for the lookup table methodology versus the traditional approach between the number of OTUs obtained when various regions of the 16S rRNA gene was used for the same biological sample. We should however take into account that perfect correspondence might be hampered by the heterogeneity of different 16S rRNA genes within the same species. Additionally, the increase in amount of OTUs—as seen for the HMP samples—was less pronounced for other environments like the rhizosphere and cooling water. For the rhizosphere and soil samples, the percentage of unclassified reads at the family level exceeded half of the total number of reads. This high percentage is forming an obstacle for our approach, as we are forced to fall back to the default 97% cut-off value instead of our family-dependent cut-off, as such reflected by the less dramatic increase in the number of OTU when compared to other types of microbiomes. The fact that a read is labelled as unclassified is due to the fact that (1) they share a high-sequence similarity with distinct taxonomic lineages, (2) they contain too many sequencing errors or (3) they belong to less intensively studied taxa. Some of these problems will most probably be solved over time via the usage of sequencing technologies producing longer read lengths with lower sequencing error rates, thereby allowing a more in-depth taxonomic classification.

In conclusion, the results within this work point out different evolutionary rates within the different variable regions of the 16S rRNA gene might have significant impact on the OTU clustering results derived from 16S rRNA amplicon sequencing data. As such caution should be taken when comparing diversity indices derived for the same environment however using different amplicons. To circumvent this problem, we developed a methodology that calculates a dynamic and evolutionary compliant OTU clustering cut-off per taxonomic family for each constructed amplicon, leading to improved OTU clustering results.

## Supplementary Material

Supplemental material Supplementary data are available at *FEMSEC* online.
