# Supplementary material for: Reconciliation between operational taxonomic units and species boundaries
Source: FEMS Microbiol Ecol. 2017 Mar 21;93(4):fix029. doi: 10.1093/femsec/fix029 (PMC5812548; doi:10.1093/femsec/fix029)
Supplement: Supplemental material — Supplementary data are available at FEMSEC online. [file fix029_supp.zip › Supplementary_file4.pdf]

					Average distances of species within the families				Percentage Improvement			
Kingdom	Phylum	Class	Order	Family	V1-V2	V3-V4	V4	V4-V5	V1-V2	V3-V4	V4	V4-V5
Bacteria	Acidobacteria	Acidobacteria	Acidobacteriales	Acidobacteriaceae	2.6%	1.5%	2.0%	1.6%	11	11	21	16
			Acidimicrobiales	Acidimicrobiaceae	8.0%	2.9%	2.8%	1.6%	0	0	0	0
			Actinomycetales	Actinomycetaceae	9.2%	2.6%	1.0%	1.6%	0	29	3	24
				Actinopolysporaceae	4.0%	0.0%	0.0%	0.3%	0	56	56	67
				Beutenbergiaceae	8.4%	1.9%	1.7%	1.0%	0	50	75	25
				Bogoriellaceae	2.5%	0.3%	0.4%	0.4%	0	50	40	50
				Brevibacteriaceae	4.8%	0.2%	0.0%	0.2%	0	48	52	48
				Catenulisporaceae	1.5%	0.5%	0.4%	0.2%	20	40	60	60
				Cellulomonadaceae	3.5%	0.4%	0.0%	0.2%	0	61	58	55
				Corynebacteriaceae	5.1%	2.1%	1.0%	1.4%	0	33	18	28
				Cryptosporangiaceae	2.9%	0.1%	0.0%	0.4%	0	50	67	67
				Demequinaceae	2.1%	0.0%	0.0%	0.2%	22	56	56	67
				Dermabacteraceae	3.1%	0.2%	0.0%	0.2%	0	56	61	78
				Dermacoccaceae	1.4%	0.3%	0.0%	0.4%	6	59	59	65
				Dermatophilaceae	8.8%	1.9%	1.7%	1.5%	0	50	33	33
				Dietziaceae	0.7%	0.4%	0.3%	0.2%	46	46	62	62
				Geodermatophilaceae	2.8%	0.4%	0.0%	0.2%	9	59	50	55
				Glycomycetaceae	0.4%	1.0%	0.3%	0.2%	6	19	25	31
				Intrasporangiaceae	4.7%	0.2%	0.0%	0.4%	0	59	63	56
				Jiangellaceae	1.3%	0.5%	0.3%	0.2%	0	60	60	60
				Kineosporiaceae	3.6%	0.4%	0.3%	0.2%	0	40	40	70
				Microbacteriaceae	6.2%	0.8%	0.7%	0.8%	0	34	34	33
				Micrococcaceae	5.7%	0.8%	0.7%	0.4%	0	40	34	48
				Micromonosporaceae	3.1%	0.4%	0.3%	0.4%	0	40	46	46
				Mycobacteriaceae	2.0%	0.4%	0.0%	0.0%	12	46	40	55
				Nakamurellaceae	8.3%	0.9%	1.4%	1.2%	0	25	25	25
				Nocardiaceae	3.1%	1.3%	1.0%	1.2%	0	26	26	26
				Nocardioidaceae	5.2%	1.0%	1.0%	0.8%	0	37	32	49
				Nocardiopsaceae	2.0%	0.8%	0.3%	0.2%	9	57	43	63
				Promicromonosporaceae	1.9%	0.6%	0.3%	0.4%	23	54	50	69
				Propionibacteriaceae	6.7%	1.9%	1.7%	1.6%	0	19	17	28
				Pseudonocardiaceae	4.8%	1.3%	1.3%	1.2%	0	19	27	22
				Sanguibacteraceae	2.7%	0.1%	0.0%	0.4%	0	50	67	83
				Streptomycetaceae	3.8%	0.2%	0.0%	0.2%	0	29	31	30
				Streptosporangiaceae	1.9%	0.2%	0.3%	0.4%	15	40	54	44
				Thermomonosporaceae	3.8%	0.8%	0.3%	0.8%	0	53	42	50
				Tsukamurellaceae	0.0%	0.0%	0.0%	0.0%	55	27	27	45
			Bifidobacteriales	Bifidobacteriaceae	5.7%	1.9%	1.0%	1.0%	0	38	16	47
			Eggerthellales	Coriobacteriaceae	9.2%	3.3%	2.7%	2.7%	0	0	0	3
			Rubrobacterales	Rubrobacteraceae	5.7%	5.5%	5.7%	4.3%	0	0	0	0
			SoliRubrobacterales	Patulibacteraceae	0.7%	2.3%	1.4%	1.0%	50	25	25	25
				Solirubrobacteraceae	3.7%	1.6%	0.2%	0.1%	0	50	25	50
	Aquificae	Aquificae	Aquificales	Aquificaceae	8.2%	2.6%	1.7%	2.4%	0	30	10	10
				Desulfurobacteriaceae	4.2%	2.9%	1.1%	2.3%	0	50	0	25
				Hydrogenothermaceae	4.3%	0.4%	0.0%	1.0%	0	22	44	22
	Bacteroidetes	Bacteroidia	Bacteroidales	Bacteroidaceae	8.6%	2.6%	2.0%	1.6%	0	13	2	13
				Marinilabiliaceae	5.6%	2.4%	0.9%	1.8%	0	0	0	0
				Porphyromonadaceae	8.9%	6.5%	5.7%	4.4%	0	0	0	0
				Prevotellaceae	8.8%	5.2%	4.4%	4.6%	0	0	0	0
				Rikenellaceae	8.9%	2.2%	1.6%	1.3%	0	25	25	25
		Cytophagia	Cytophagales	Cyclobacteriaceae	5.7%	1.2%	0.7%	1.4%	0	31	25	20
				Cytophagaceae	9.9%	3.2%	3.0%	3.2%	0	0	0	0
				Flammeovirgaceae	9.0%	3.1%	3.4%	2.2%	0	0	0	6
				Rhodothermaceae	5.4%	4.9%	4.6%	3.9%	0	0	0	0
		Flavobacteriia	Flavobacteriales	Cryomorphaceae	10.6%	4.6%	4.7%	3.9%	0	0	0	0
				Flavobacteriaceae	8.6%	4.3%	3.7%	3.4%	0	0	0	0
		Sphingobacteriia	Sphingobacteriales	Chitinophagaceae	7.0%	3.4%	3.0%	2.6%	0	0	0	9
				Saprospiraceae	14.6%	5.5%	2.7%	3.0%	0	10	0	0
				Sphingobacteriaceae	6.0%	2.4%	1.7%	1.4%	0	22	11	26
	Chlamydiae	Chlamydiae	Chlamydiales	Chlamydiaceae	2.3%	0.7%	0.0%	0.0%	11	33	56	56
	Chlorobi	Chlorobia	Chlorobiales	Chlorobiaceae	4.9%	2.9%	1.7%	1.1%	0	27	9	36
	Chloroflexi	Anaerolineae	Anaerolineales	Anaerolineaceae	10.0%	6.0%	7.5%	6.5%	0	0	0	0
		Caldilineae	Caldilineales	Caldilineaceae	23.6%	6.3%	7.1%	5.6%	0	0	0	0
		Dehalococcoidia	Dehalococcoidales	Dehalococcoidaceae	8.0%	2.9%	3.8%	2.9%	0	0	0	0
		Ktedonobacteria	Thermogemmatisporales	Thermogemmatisporaceae	13.4%	1.1%	0.7%	0.4%	0	33	67	33
	Chrysiogenetes	Chrysiogenetes	Chrysiogenales	Chrysiogenaceae	1.6%	0.6%	0.5%	0.4%	0	0	0	0
	Deferribacteres	Deferribacteres	Deferribacterales	Deferribacteraceae	4.7%	2.5%	2.1%	1.6%	0	0	0	9
	Deinococcus-Thermus	Deinococci	Deinococcales	Deinococcaceae	8.0%	2.9%	2.7%	2.0%	0	6	0	13
			Thermales	Thermaceae	6.0%	1.7%	1.7%	1.6%	0	21	21	21
	Fibrobacteres	Fibrobacteria	Fibrobacterales	Fibrobacteraceae	6.1%	5.6%	4.5%	4.8%	0	0	0	0
	Firmicutes	Bacilli	Bacillales	Alicyclobacillaceae	6.5%	1.8%	2.0%	1.4%	0	19	11	15
				Bacillaceae	8.8%	3.0%	1.7%	1.8%	0	20	0	16
				Listeriaceae	1.5%	0.0%	0.0%	0.0%	29	21	29	29
				Paenibacillaceae	9.0%	2.8%	2.4%	1.6%	0	12	4	22
				Planococcaceae	4.2%	0.8%	0.3%	0.4%	0	46	40	49
				Sporolactobacillaceae	2.8%	1.0%	0.2%	0.6%	0	50	42	50
				Staphylococcaceae	2.3%	0.6%	0.0%	0.0%	13	44	33	49
				Thermoactinomycetaceae	4.0%	2.1%	1.0%	1.1%	0	22	11	30
			Lactobacillales	Aerococcaceae	9.6%	3.4%	2.0%	1.6%	0	28	0	17
				Carnobacteriaceae	3.5%	0.4%	0.0%	0.2%	0	34	27	46
				Enterococcaceae	1.5%	0.4%	0.0%	0.0%	10	46	59	60
				Lactobacillaceae	10.3%	2.4%	1.7%	1.4%	0	21	7	22
				Leuconostocaceae	2.8%	0.6%	0.3%	0.4%	2	40	43	48
				Streptococcaceae	6.6%	1.4%	0.7%	1.0%	0	28	23	31
		Clostridia	Clostridiales	Caldicoprobacteraceae	14.0%	0.3%	0.0%	0.1%	0	33	33	33
				Clostridiaceae	9.4%	3.8%	4.4%	4.0%	0	0	0	0
				Eubacteriaceae	9.8%	3.4%	3.7%	3.1%	0	0	0	0
				Heliobacteriaceae	5.5%	2.3%	1.3%	1.5%	0	11	22	33
				Lachnospiraceae	8.5%	3.3%	4.0%	3.9%	0	0	0	0
				Peptococcaceae	9.5%	3.0%	2.6%	2.2%	0	7	0	5
				Peptostreptococcaceae	10.0%	1.3%	1.5%	1.7%	0	18	27	36
				Ruminococcaceae	12.7%	4.9%	5.1%	4.3%	0	0	0	0
				Syntrophomonadaceae	12.5%	5.9%	3.7%	2.9%	0	0	0	0
			Halanaerobiales	Halanaerobiaceae	1.2%	0.4%	0.0%	0.0%	31	38	31	46
				Halobacteroidaceae	17.0%	6.3%	3.7%	3.3%	0	0	0	0
			Natranaerobiales	Natranaerobiaceae	14.9%	2.6%	3.6%	3.6%	0	0	0	0
			Thermoanaerobacterales	Thermoanaerobacteraceae	3.8%	1.5%	1.3%	1.0%	0	21	23	30
				Thermodesulfobiaceae	31.8%	20.1%	20.1%	17.2%	0	0	0	0
		Erysipelotrichia	Erysipelotrichales	Erysipelotrichaceae	7.2%	2.7%	1.0%	1.2%	0	0	0	0
		Negativicutes	Selenomonadales	Acidaminococcaceae	14.2%	6.5%	4.6%	3.5%	0	0	0	0
				Veillonellaceae	7.2%	3.6%	2.3%	1.8%	0	4	0	18
	Fusobacteria	Fusobacteriia	Fusobacteriales	Fusobacteriaceae	3.8%	1.3%	0.7%	0.8%	0	36	29	32
				Leptotrichiaceae	6.1%	1.3%	1.7%	1.3%	0	30	20	30
	Nitrospira	Nitrospira	Nitrospirales	Nitrospiraceae	3.8%	2.1%	2.7%	2.5%	0	0	13	13
	Planctomycetes	Planctomycetacia	Planctomycetales	Planctomycetaceae	13.5%	7.0%	8.7%	7.3%	0	0	0	0
	Proteobacteria	Acidithiobacillia	Acidithiobacillales	Acidithiobacillaceae	1.0%	0.5%	0.2%	0.2%	17	50	50	50
		Alphaproteobacteria	Caulobacterales	Caulobacteraceae	1.5%	0.8%	1.0%	1.0%	31	33	47	42
				Hyphomonadaceae	3.1%	1.5%	2.0%	1.2%	0	10	26	26
			Kordiimonadales	Kordiimonadaceae	4.5%	1.7%	2.1%	2.0%	0	25	50	50
			Parvularculales	Parvularculaceae	4.3%	4.0%	3.4%	3.7%	0	0	0	0
			Rhizobiales	Aurantimonadaceae	2.1%	1.1%	0.7%	1.3%	17	42	42	33
				Bartonellaceae	0.4%	0.0%	0.0%	0.0%	50	43	47	60
				Beijerinckiaceae	1.3%	0.0%	0.0%	0.1%	15	55	60	65
				Bradyrhizobiaceae	0.9%	0.0%	0.0%	0.0%	41	35	53	57
				Brucellaceae	0.0%	0.0%	0.0%	0.0%	32	34	42	45
				Cohaesibacteraceae	3.1%	1.9%	2.0%	1.2%	0	0	33	33
				Hyphomicrobiaceae	3.8%	1.5%	1.7%	1.6%	0	16	23	23
				Methylobacteriaceae	2.2%	0.2%	0.0%	0.2%	5	47	53	61
				Methylocystaceae	2.1%	0.2%	0.0%	0.8%	10	60	50	60
				Phyllobacteriaceae	0.5%	0.4%	0.0%	0.2%	34	49	48	45
				Rhizobiaceae	1.2%	0.0%	0.0%	0.4%	23	48	58	48
				Rhodobiaceae	4.4%	2.2%	2.5%	2.2%	0	0	7	7
				Xanthobacteraceae	2.0%	0.4%	0.5%	0.4%	18	50	57	46
			Rhodobacterales	Rhodobacteraceae	4.9%	2.3%	2.3%	2.2%	0	11	14	15
			Rhodospirillales	Acetobacteraceae	1.5%	1.1%	1.0%	1.2%	14	26	27	26
				Rhodospirillaceae	6.9%	2.3%	2.4%	2.0%	0	8	4	6
			Rickettsiales	Anaplasmataceae	5.3%	0.8%	0.9%	0.5%	0	38	38	38
				Rickettsiaceae	1.1%	0.0%	0.0%	0.2%	55	55	60	65
			Sneathiellales	Sneathiellaceae	6.4%	2.8%	2.8%	1.9%	0	0	0	20
			Sphingomonadales	Erythrobacteraceae	1.3%	0.8%	0.7%	0.8%	30	49	49	46
				Sphingomonadaceae	3.7%	1.3%	1.7%	1.8%	0	25	37	21
		Betaproteobacteria	Burkholderiales	Alcaligenaceae	1.0%	1.0%	0.3%	0.4%	21	40	37	48
				Burkholderiaceae	1.5%	1.6%	0.3%	0.4%	22	41	34	43
				Comamonadaceae	3.1%	2.4%	1.0%	1.2%	0	38	13	42
				Oxalobacteraceae	2.6%	1.8%	0.3%	0.4%	1	43	28	48
				Sutterellaceae	7.3%	4.9%	2.9%	2.7%	0	0	0	0
			Hydrogenophilales	Hydrogenophilaceae	1.5%	1.2%	0.8%	1.0%	18	27	27	36
			Methylophilales	Methylophilaceae	1.0%	0.4%	0.0%	0.0%	31	44	44	56
			Neisseriales	Chromobacteriaceae	0.2%	0.2%	0.3%	0.2%	57	57	57	71
				Neisseriaceae	5.6%	3.4%	2.0%	2.0%	0	12	0	20
			Nitrosomonadales	Nitrosomonadaceae	8.9%	7.0%	6.4%	5.6%	0	0	0	0
			Rhodocyclales	Rhodocyclaceae	4.1%	2.4%	0.7%	0.8%	0	35	6	35
		Deltaproteobacteria	Bdellovibrionales	Bacteriovoracaceae	19.1%	10.3%	7.9%	6.7%	0	0	0	0
				Bdellovibrionaceae	31.6%	24.6%	23.2%	19.5%	0	0	0	0
			Desulfobacterales	Desulfobacteraceae	5.4%	3.8%	2.0%	1.8%	0	13	0	6
				Desulfobulbaceae	7.4%	3.6%	2.7%	2.1%	0	6	0	0
				Desulfohalobiaceae	11.5%	2.2%	1.0%	1.9%	0	38	8	31
				Desulfomicrobiaceae	0.2%	0.1%	0.2%	0.2%	29	43	43	57
				Desulfonatronaceae	1.3%	1.1%	0.3%	0.9%	29	43	43	57
				Desulfovibrionaceae	6.9%	4.2%	3.0%	3.0%	0	0	0	0
			Desulfurellales	Desulfurellaceae	0.3%	0.1%	0.2%	0.4%	29	43	29	57
			Desulfuromonadales	Desulfuromonadaceae	5.3%	1.6%	1.3%	1.0%	0	47	21	42
				Geobacteraceae	4.0%	1.9%	1.0%	1.0%	0	36	23	36
			Myxococcales	Cystobacteraceae	0.0%	0.1%	0.0%	0.0%	41	53	59	59
				Myxococcaceae	0.2%	0.1%	0.0%	0.1%	71	43	43	57
				Nannocystaceae	2.6%	0.8%	0.7%	0.6%	0	20	40	40
				Polyangiaceae	1.1%	0.2%	0.0%	0.3%	9	45	27	64
			Syntrophobacterales	Syntrophaceae	4.8%	1.2%	1.3%	1.0%	0	14	14	14
				Syntrophobacteraceae	8.6%	5.1%	3.1%	2.0%	0	0	0	17
		Epsilonproteobacteria	Campylobacterales	Campylobacteraceae	3.0%	1.1%	1.0%	1.2%	0	24	35	25
				Helicobacteraceae	1.1%	1.1%	1.3%	1.1%	33	33	45	45
			Nautiliales	Nautiliaceae	6.2%	0.8%	0.9%	1.3%	0	18	18	18
		Gammaproteobacteria	Aeromonadales	Aeromonadaceae	0.0%	0.2%	0.0%	0.0%	49	33	39	39
				Succinivibrionaceae	10.1%	8.3%	9.6%	7.9%	0	0	0	0
			Alteromonadales	Alteromonadaceae	4.1%	2.2%	0.7%	1.0%	0	36	17	34
				Colwelliaceae	2.4%	2.0%	0.7%	1.0%	5	43	29	43
				Ferrimonadaceae	3.4%	0.6%	0.6%	1.0%	0	44	33	44
				Idiomarinaceae	1.7%	1.4%	0.3%	0.6%	4	54	33	63
				Moritellaceae	0.0%	1.1%	0.0%	0.0%	22	33	56	44
				Pseudoalteromonadaceae	0.7%	0.0%	0.0%	0.3%	30	36	59	55
				Psychromonadaceae	1.9%	1.5%	0.0%	0.0%	14	43	29	64
				Shewanellaceae	1.1%	1.8%	0.3%	0.4%	18	39	23	46
			Cardiobacteriales	Cardiobacteriaceae	12.4%	5.3%	3.2%	3.1%	0	0	0	0
			Chromatiales	Chromatiaceae	3.6%	2.8%	2.0%	1.6%	0	12	3	17
				Ectothiorhodospiraceae	4.6%	4.2%	3.7%	3.3%	0	0	0	0
				Halothiobacillaceae	10.8%	3.7%	4.2%	3.5%	0	0	0	0
				Thioalkalispiraceae	5.3%	0.9%	0.7%	1.1%	0	0	0	0
			Enterobacteriales	Enterobacteriaceae	2.3%	1.6%	0.3%	0.6%	12	42	24	36
			Legionellales	Coxiellaceae	22.5%	13.8%	15.5%	14.7%	0	0	0	0
				Legionellaceae	3.7%	1.8%	1.0%	1.0%	0	31	22	35
			Lysobacteriales	Lysobacteraceae	2.7%	1.6%	1.3%	1.0%	3	27	27	37
				Nevskiaceae	2.6%	3.5%	2.8%	2.6%	0	0	0	14
				Sinobacteraceae	4.0%	1.2%	0.4%	0.3%	0	50	33	50
			Methylococcales	Methylococcaceae	2.1%	2.2%	2.0%	2.0%	14	17	14	11
			Oceanospirillales	Alcanivoracaceae	2.3%	1.4%	0.7%	0.4%	13	63	38	50
				Hahellaceae	5.9%	2.0%	1.7%	1.8%	0	23	15	31
				Halomonadaceae	2.7%	1.4%	0.7%	0.4%	5	39	30	45
				Litoricolaceae	4.3%	2.8%	1.9%	3.8%	0	0	0	0
				Oceanospirillaceae	4.6%	2.8%	1.0%	0.9%	0	21	5	34
				Saccharospirillaceae	4.3%	2.2%	1.0%	1.0%	0	33	33	33
			Orbales	Orbaceae	6.6%	3.1%	1.2%	3.9%	0	0	0	0
			Pasteurellales	Pasteurellaceae	5.3%	3.0%	1.3%	1.4%	0	26	0	32
			Pseudomonadales	Moraxellaceae	2.6%	1.6%	0.7%	0.8%	5	40	27	33
				Pseudomonadaceae	0.7%	1.2%	0.3%	0.6%	32	34	22	36
			Salinisphaerales	Salinisphaeraceae	3.1%	1.0%	0.8%	1.2%	0	43	43	14
			Thiotrichales	Francisellaceae	0.3%	0.2%	0.0%	0.2%	44	44	56	67
				Piscirickettsiaceae	4.1%	2.0%	1.0%	0.8%	0	34	17	38
				Thiotrichaceae	2.1%	0.9%	1.2%	1.2%	8	0	0	0
			Vibrionales	Vibrionaceae	1.8%	1.6%	0.7%	0.4%	18	40	28	50
	Spirochaetes	Spirochaetes	Spirochaetales	Brachyspiraceae	0.7%	0.2%	0.3%	0.2%	57	57	71	57
				Leptospiraceae	0.7%	0.0%	0.0%	0.0%	39	35	43	39
				Spirochaetaceae	3.0%	1.6%	1.7%	1.4%	0	14	18	9
	Synergistetes	Synergistia	Synergistales	Synergistaceae	3.6%	1.7%	1.7%	2.0%	0	14	10	5
	Tenericutes	Mollicutes	Acholeplasmatales	Acholeplasmataceae	5.7%	3.5%	2.9%	2.5%	0	0	0	0
			Anaeroplasmatales	Anaeroplasmataceae	4.3%	2.9%	2.7%	2.0%	0	0	0	0
			Entomoplasmatales	Entomoplasmataceae	0.6%	0.0%	0.0%	0.0%	31	31	38	38
				Spiroplasmataceae	1.0%	0.8%	1.0%	0.8%	31	34	43	40
			Mycoplasmatales	Mycoplasmataceae	8.0%	3.6%	2.0%	2.4%	0	15	0	8
	Thermodesulfobacteria	Thermodesulfobacteria	Thermodesulfobacteriales	Thermodesulfobacteriaceae	1.8%	2.4%	1.7%	1.7%	13	50	0	38
	Thermotogae	Thermotogae	Kosmotogales	Kosmotogaceae	10.8%	0.2%	0.2%	0.1%	0	0	0	0
			Petrotogales	Petrotogaceae	1.3%	0.4%	0.1%	0.1%	17	67	50	67
			Thermotogales	Fervidobacteriaceae	3.1%	1.7%	2.0%	2.7%	0	0	20	20
				Thermotogaceae	2.8%	1.0%	1.1%	1.2%	4	29	18	25
	Verrucomicrobia	Opitutae	Puniceicoccales	Puniceicoccaceae	4.5%	2.6%	0.8%	1.6%	0	29	0	29
		Spartobacteria	Spartobacteria	Verrucomicrobiaceae	5.0%	3.0%	1.7%	1.6%	0	15	0	27
		Verrucomicrobiae	Verrucomicrobiales	Rubritaleaceae	1.2%	0.6%	0.3%	0.2%	33	50	33	50
Archaea	Crenarchaeota	Thermoprotei	Desulfurococcales	Desulfurococcaceae	15.7%	13.1%	6.0%	7.2%	0	0	0	0
			Sulfolobales	Sulfolobaceae	3.8%	5.9%	5.2%	6.3%	0	0	0	0
			Thermoproteales	Thermoproteaceae	11.2%	1.8%	1.4%	3.0%	0	0	10	0
	Euryarchaeota	Archaeoglobi	Archaeoglobales	Archaeoglobaceae	17.9%	7.9%	4.7%	3.7%	0	0	0	0
		Halobacteria	Halobacteriales	Halobacteriaceae	19.0%	13.1%	8.2%	7.3%	0	0	0	0
		Methanobacteria	Methanobacteriales	Methanobacteriaceae	11.2%	5.2%	3.8%	3.7%	0	0	0	0
		Methanococci	Methanococcales	Methanocaldococcaceae	17.2%	6.7%	2.0%	3.7%	0	13	0	0
				Methanococcaceae	16.8%	22.1%	6.1%	7.4%	0	0	0	0
		Methanomicrobia	Methanocellales	Methanocellaceae	29.6%	9.8%	3.7%	7.1%	0	0	0	0
			Methanomicrobiales	Methanocorpusculaceae	23.7%	1.5%	1.5%	2.1%	0	0	0	0
				Methanomicrobiaceae	4.9%	3.4%	3.3%	3.3%	0	0	0	0
				Methanoregulaceae	14.6%	10.1%	5.8%	5.9%	0	0	0	0
				Methanospirillaceae	30.8%	7.5%	3.6%	2.6%	0	0	0	0
			Methanosarcinales	Methanosarcinaceae	13.1%	3.7%	2.0%	2.0%	0	10	0	14
				Methanotrichaceae	69.5%	34.6%	9.5%	10.1%	0	0	0	0
		Thermococci	Thermococcales	Thermococcaceae	1.2%	0.0%	0.0%	0.2%	14	47	42	56
		Thermoplasmata	Thermoplasmatales	Ferroplasmaceae	100.0%	100.0%	100.0%	100.0%	0	0	0	0

Kingdom	Phylum	Class	order	Family	Average distances of species within the families: V1-V2	V3-V4	V4	V4-V5	Percentage Improvement: V1-V2	V3-V4	V4	V4-V5
Bacteria	Acidobacteria	Acidobacteria	Acidobacteriales	Acidobacteriaceae	2.6%	1.5%	2.0%	1.6%	11	11	21	16
			Acidimicrobiales	Acidimicrobiaceae	8.0%	2.9%	2.8%	1.6%	0	0	0	0
			Actinomycetales	Actinomycetaceae	9.2%	2.6%	1.0%	1.6%	0	29	3	24
				Actinopolysporaceae	4.0%	0.0%	0.0%	0.3%	0	56	56	67
				Beutenbergiaceae	8.4%	1.9%	1.7%	1.0%	0	50	75	25
				Bogoriellaceae	2.5%	0.3%	0.4%	0.4%	0	50	40	50
				Brevibacteriaceae	4.8%	0.2%	0.0%	0.2%	0	48	52	48
				Catenulisporaceae	1.5%	0.5%	0.4%	0.2%	20	40	60	60
				Cellulomonadaceae	3.5%	0.4%	0.0%	0.2%	0	61	58	55
				Corynebacteriaceae	5.1%	2.1%	1.0%	1.4%	0	33	18	28
				Cryptosporangiaceae	2.9%	0.1%	0.0%	0.4%	0	50	67	67
				Demequinaceae	2.1%	0.0%	0.0%	0.2%	22	56	56	67
				Dermabacteraceae	3.1%	0.2%	0.0%	0.2%	0	56	61	78
				Dermacoccaceae	1.4%	0.3%	0.0%	0.4%	6	59	59	65
				Dermatophilaceae	8.8%	1.9%	1.7%	1.5%	0	50	33	33
				Dietziaceae	0.7%	0.4%	0.3%	0.2%	46	46	62	62
				Geodermatophilaceae	2.8%	0.4%	0.0%	0.2%	9	59	50	55
				Glycomycetaceae	0.4%	1.0%	0.3%	0.2%	6	19	25	31
				Intrasporangiaceae	4.7%	0.2%	0.0%	0.4%	0	59	63	56
				Jiangellaceae	1.3%	0.5%	0.3%	0.2%	0	60	60	60
				Kineosporiaceae	3.6%	0.4%	0.3%	0.2%	0	40	40	70
				Microbacteriaceae	6.2%	0.8%	0.7%	0.8%	0	34	34	33
				Micrococcaceae	5.7%	0.8%	0.7%	0.4%	0	40	34	48
				Micromonosporaceae	3.1%	0.4%	0.3%	0.4%	0	40	46	46
				Mycobacteriaceae	2.0%	0.4%	0.0%	0.0%	12	46	40	55
				Nakamurellaceae	8.3%	0.9%	1.4%	1.2%	0	25	25	25
				Nocardiaceae	3.1%	1.3%	1.0%	1.2%	0	26	26	26
				Nocardioidaceae	5.2%	1.0%	1.0%	0.8%	0	37	32	49
				Nocardiopsaceae	2.0%	0.8%	0.3%	0.2%	9	57	43	63
				Promicromonosporaceae	1.9%	0.6%	0.3%	0.4%	23	54	50	69
				Propionibacteriaceae	6.7%	1.9%	1.7%	1.6%	0	19	17	28
				Pseudonocardiaceae	4.8%	1.3%	1.3%	1.2%	0	19	27	22
				Sanguibacteraceae	2.7%	0.1%	0.0%	0.4%	0	50	67	83
				Streptomycetaceae	3.8%	0.2%	0.0%	0.2%	0	29	31	30
				Streptosporangiaceae	1.9%	0.2%	0.3%	0.4%	15	40	54	44
				Thermomonosporaceae	3.8%	0.8%	0.3%	0.8%	0	53	42	50
				Tsukamurellaceae	0.0%	0.0%	0.0%	0.0%	55	27	27	45
			Bifidobacteriales	Bifidobacteriaceae	5.7%	1.9%	1.0%	1.0%	0	38	16	47
			Eggerthellales	Coriobacteriaceae	9.2%	3.3%	2.7%	2.7%	0	0	0	3
			Rubrobacterales	Rubrobacteraceae	5.7%	5.5%	5.7%	4.3%	0	0	0	0
			SoliRubrobacterales	Patulibacteraceae	0.7%	2.3%	1.4%	1.0%	50	25	25	25
				Solirubrobacteraceae	3.7%	1.6%	0.2%	0.1%	0	50	25	50
	Aquificae	Aquificae	Aquificales	Aquificaceae	8.2%	2.6%	1.7%	2.4%	0	30	10	10
				Desulfurobacteriaceae	4.2%	2.9%	1.1%	2.3%	0	50	0	25
				Hydrogenothermaceae	4.3%	0.4%	0.0%	1.0%	0	22	44	22
	Bacteroidetes	Bacteroidia	Bacteroidales	Bacteroidaceae	8.6%	2.6%	2.0%	1.6%	0	13	2	13
				Marinilabiliaceae	5.6%	2.4%	0.9%	1.8%	0	0	0	0
				Porphyromonadaceae	8.9%	6.5%	5.7%	4.4%	0	0	0	0
				Prevotellaceae	8.8%	5.2%	4.4%	4.6%	0	0	0	0
				Rikenellaceae	8.9%	2.2%	1.6%	1.3%	0	25	25	25
		Cytophagia	Cytophagales	Cyclobacteriaceae	5.7%	1.2%	0.7%	1.4%	0	31	25	20
				Cytophagaceae	9.9%	3.2%	3.0%	3.2%	0	0	0	0
				Flammeovirgaceae	9.0%	3.1%	3.4%	2.2%	0	0	0	6
				Rhodothermaceae	5.4%	4.9%	4.6%	3.9%	0	0	0	0
		Flavobacteriia	Flavobacteriales	Cryomorphaceae	10.6%	4.6%	4.7%	3.9%	0	0	0	0
				Flavobacteriaceae	8.6%	4.3%	3.7%	3.4%	0	0	0	0
		Sphingobacteriia	Sphingobacteriales	Chitinophagaceae	7.0%	3.4%	3.0%	2.6%	0	0	0	9
				Saprospiraceae	14.6%	5.5%	2.7%	3.0%	0	10	0	0
				Sphingobacteriaceae	6.0%	2.4%	1.7%	1.4%	0	22	11	26
	Chlamydiae	Chlamydiae	Chlamydiales	Chlamydiaceae	2.3%	0.7%	0.0%	0.0%	11	33	56	56
	Chlorobi	Chlorobia	Chlorobiales	Chlorobiaceae	4.9%	2.9%	1.7%	1.1%	0	27	9	36
	Chloroflexi	Anaerolineae	Anaerolineales	Anaerolineaceae	10.0%	6.0%	7.5%	6.5%	0	0	0	0
		Caldilineae	Caldilineales	Caldilineaceae	23.6%	6.3%	7.1%	5.6%	0	0	0	0
		Dehalococcoidia	Dehalococcoidales	Dehalococcoidaceae	8.0%	2.9%	3.8%	2.9%	0	0	0	0
		Ktedonobacteria	Thermogemmatisporales	Thermogemmatisporaceae	13.4%	1.1%	0.7%	0.4%	0	33	67	33
	Chrysiogenetes	Chrysiogenetes	Chrysiogenales	Chrysiogenaceae	1.6%	0.6%	0.5%	0.4%	0	0	0	0
	Deferribacteres	Deferribacteres	Deferribacterales	Deferribacteraceae	4.7%	2.5%	2.1%	1.6%	0	0	0	9
	Deinococcus-Thermus	Deinococci	Deinococcales	Deinococcaceae	8.0%	2.9%	2.7%	2.0%	0	6	0	13
			Thermales	Thermaceae	6.0%	1.7%	1.7%	1.6%	0	21	21	21
	Fibrobacteres	Fibrobacteria	Fibrobacterales	Fibrobacteraceae	6.1%	5.6%	4.5%	4.8%	0	0	0	0
	Firmicutes	Bacilli	Bacillales	Alicyclobacillaceae	6.5%	1.8%	2.0%	1.4%	0	19	11	15
				Bacillaceae	8.8%	3.0%	1.7%	1.8%	0	20	0	16
				Listeriaceae	1.5%	0.0%	0.0%	0.0%	29	21	29	29
				Paenibacillaceae	9.0%	2.8%	2.4%	1.6%	0	12	4	22
				Planococcaceae	4.2%	0.8%	0.3%	0.4%	0	46	40	49
				Sporolactobacillaceae	2.8%	1.0%	0.2%	0.6%	0	50	42	50
				Staphylococcaceae	2.3%	0.6%	0.0%	0.0%	13	44	33	49
				Thermoactinomycetaceae	4.0%	2.1%	1.0%	1.1%	0	22	11	30
			Lactobacillales	Aerococcaceae	9.6%	3.4%	2.0%	1.6%	0	28	0	17
				Carnobacteriaceae	3.5%	0.4%	0.0%	0.2%	0	34	27	46
				Enterococcaceae	1.5%	0.4%	0.0%	0.0%	10	46	59	60
				Lactobacillaceae	10.3%	2.4%	1.7%	1.4%	0	21	7	22
				Leuconostocaceae	2.8%	0.6%	0.3%	0.4%	2	40	43	48
				Streptococcaceae	6.6%	1.4%	0.7%	1.0%	0	28	23	31
		Clostridia	Clostridiales	Caldicoprobacteraceae	14.0%	0.3%	0.0%	0.1%	0	33	33	33
				Clostridiaceae	9.4%	3.8%	4.4%	4.0%	0	0	0	0
				Eubacteriaceae	9.8%	3.4%	3.7%	3.1%	0	0	0	0
				Heliobacteriaceae	5.5%	2.3%	1.3%	1.5%	0	11	22	33
				Lachnospiraceae	8.5%	3.3%	4.0%	3.9%	0	0	0	0
				Peptococcaceae	9.5%	3.0%	2.6%	2.2%	0	7	0	5
				Peptostreptococcaceae	10.0%	1.3%	1.5%	1.7%	0	18	27	36
				Ruminococcaceae	12.7%	4.9%	5.1%	4.3%	0	0	0	0
				Syntrophomonadaceae	12.5%	5.9%	3.7%	2.9%	0	0	0	0
			Halanaerobiales	Halanaerobiaceae	1.2%	0.4%	0.0%	0.0%	31	38	31	46
				Halobacteroidaceae	17.0%	6.3%	3.7%	3.3%	0	0	0	0
			Natranaerobiales	Natranaerobiaceae	14.9%	2.6%	3.6%	3.6%	0	0	0	0
			Thermoanaerobacterales	Thermoanaerobacteraceae	3.8%	1.5%	1.3%	1.0%	0	21	23	30
				Thermodesulfobiaceae	31.8%	20.1%	20.1%	17.2%	0	0	0	0
		Erysipelotrichia	Erysipelotrichales	Erysipelotrichaceae	7.2%	2.7%	1.0%	1.2%	0	0	0	0
		Negativicutes	Selenomonadales	Acidaminococcaceae	14.2%	6.5%	4.6%	3.5%	0	0	0	0
				Veillonellaceae	7.2%	3.6%	2.3%	1.8%	0	4	0	18
	Fusobacteria	Fusobacteriia	Fusobacteriales	Fusobacteriaceae	3.8%	1.3%	0.7%	0.8%	0	36	29	32
				Leptotrichiaceae	6.1%	1.3%	1.7%	1.3%	0	30	20	30
	Nitrospira	Nitrospira	Nitrospirales	Nitrospiraceae	3.8%	2.1%	2.7%	2.5%	0	0	13	13
	Planctomycetes	Planctomycetacia	Planctomycetales	Planctomycetaceae	13.5%	7.0%	8.7%	7.3%	0	0	0	0
	Proteobacteria	Acidithiobacillia	Acidithiobacillales	Acidithiobacillaceae	1.0%	0.5%	0.2%	0.2%	17	50	50	50
		Alphaproteobacteria	Caulobacterales	Caulobacteraceae	1.5%	0.8%	1.0%	1.0%	31	33	47	42
				Hyphomonadaceae	3.1%	1.5%	2.0%	1.2%	0	10	26	26
			Kordiimonadales	Kordiimonadaceae	4.5%	1.7%	2.1%	2.0%	0	25	50	50
			Parvularculales	Parvularculaceae	4.3%	4.0%	3.4%	3.7%	0	0	0	0
			Rhizobiales	Aurantimonadaceae	2.1%	1.1%	0.7%	1.3%	17	42	42	33
				Bartonellaceae	0.4%	0.0%	0.0%	0.0%	50	43	47	60
				Beijerinckiaceae	1.3%	0.0%	0.0%	0.1%	15	55	60	65
				Bradyrhizobiaceae	0.9%	0.0%	0.0%	0.0%	41	35	53	57
				Brucellaceae	0.0%	0.0%	0.0%	0.0%	32	34	42	45
				Cohaesibacteraceae	3.1%	1.9%	2.0%	1.2%	0	0	33	33
				Hyphomicrobiaceae	3.8%	1.5%	1.7%	1.6%	0	16	23	23
				Methylobacteriaceae	2.2%	0.2%	0.0%	0.2%	5	47	53	61
				Methylocystaceae	2.1%	0.2%	0.0%	0.8%	10	60	50	60
				Phyllobacteriaceae	0.5%	0.4%	0.0%	0.2%	34	49	48	45
				Rhizobiaceae	1.2%	0.0%	0.0%	0.4%	23	48	58	48
				Rhodobiaceae	4.4%	2.2%	2.5%	2.2%	0	0	7	7
				Xanthobacteraceae	2.0%	0.4%	0.5%	0.4%	18	50	57	46
			Rhodobacterales	Rhodobacteraceae	4.9%	2.3%	2.3%	2.2%	0	11	14	15
			Rhodospirillales	Acetobacteraceae	1.5%	1.1%	1.0%	1.2%	14	26	27	26
				Rhodospirillaceae	6.9%	2.3%	2.4%	2.0%	0	8	4	6
			Rickettsiales	Anaplasmataceae	5.3%	0.8%	0.9%	0.5%	0	38	38	38
				Rickettsiaceae	1.1%	0.0%	0.0%	0.2%	55	55	60	65
			Sneathiellales	Sneathiellaceae	6.4%	2.8%	2.8%	1.9%	0	0	0	20
			Sphingomonadales	Erythrobacteraceae	1.3%	0.8%	0.7%	0.8%	30	49	49	46
				Sphingomonadaceae	3.7%	1.3%	1.7%	1.8%	0	25	37	21
		Betaproteobacteria	Burkholderiales	Alcaligenaceae	1.0%	1.0%	0.3%	0.4%	21	40	37	48
				Burkholderiaceae	1.5%	1.6%	0.3%	0.4%	22	41	34	43
				Comamonadaceae	3.1%	2.4%	1.0%	1.2%	0	38	13	42
				Oxalobacteraceae	2.6%	1.8%	0.3%	0.4%	1	43	28	48
				Sutterellaceae	7.3%	4.9%	2.9%	2.7%	0	0	0	0
			Hydrogenophilales	Hydrogenophilaceae	1.5%	1.2%	0.8%	1.0%	18	27	27	36
			Methylophilales	Methylophilaceae	1.0%	0.4%	0.0%	0.0%	31	44	44	56
			Neisseriales	Chromobacteriaceae	0.2%	0.2%	0.3%	0.2%	57	57	57	71
				Neisseriaceae	5.6%	3.4%	2.0%	2.0%	0	12	0	20
			Nitrosomonadales	Nitrosomonadaceae	8.9%	7.0%	6.4%	5.6%	0	0	0	0
			Rhodocyclales	Rhodocyclaceae	4.1%	2.4%	0.7%	0.8%	0	35	6	35
		Deltaproteobacteria	Bdellovibrionales	Bacteriovoracaceae	19.1%	10.3%	7.9%	6.7%	0	0	0	0
				Bdellovibrionaceae	31.6%	24.6%	23.2%	19.5%	0	0	0	0
			Desulfobacterales	Desulfobacteraceae	5.4%	3.8%	2.0%	1.8%	0	13	0	6
				Desulfobulbaceae	7.4%	3.6%	2.7%	2.1%	0	6	0	0
				Desulfohalobiaceae	11.5%	2.2%	1.0%	1.9%	0	38	8	31
				Desulfomicrobiaceae	0.2%	0.1%	0.2%	0.2%	29	43	43	57
				Desulfonatronaceae	1.3%	1.1%	0.3%	0.9%	29	43	43	57
				Desulfovibrionaceae	6.9%	4.2%	3.0%	3.0%	0	0	0	0
			Desulfurellales	Desulfurellaceae	0.3%	0.1%	0.2%	0.4%	29	43	29	57
			Desulfuromonadales	Desulfuromonadaceae	5.3%	1.6%	1.3%	1.0%	0	47	21	42
				Geobacteraceae	4.0%	1.9%	1.0%	1.0%	0	36	23	36
			Myxococcales	Cystobacteraceae	0.0%	0.1%	0.0%	0.0%	41	53	59	59
				Myxococcaceae	0.2%	0.1%	0.0%	0.1%	71	43	43	57
				Nannocystaceae	2.6%	0.8%	0.7%	0.6%	0	20	40	40
				Polyangiaceae	1.1%	0.2%	0.0%	0.3%	9	45	27	64
			Syntrophobacterales	Syntrophaceae	4.8%	1.2%	1.3%	1.0%	0	14	14	14
				Syntrophobacteraceae	8.6%	5.1%	3.1%	2.0%	0	0	0	17
		Epsilonproteobacteria	Campylobacterales	Campylobacteraceae	3.0%	1.1%	1.0%	1.2%	0	24	35	25
				Helicobacteraceae	1.1%	1.1%	1.3%	1.1%	33	33	45	45
			Nautiliales	Nautiliaceae	6.2%	0.8%	0.9%	1.3%	0	18	18	18
		Gammaproteobacteria	Aeromonadales	Aeromonadaceae	0.0%	0.2%	0.0%	0.0%	49	33	39	39
				Succinivibrionaceae	10.1%	8.3%	9.6%	7.9%	0	0	0	0
			Alteromonadales	Alteromonadaceae	4.1%	2.2%	0.7%	1.0%	0	36	17	34
				Colwelliaceae	2.4%	2.0%	0.7%	1.0%	5	43	29	43
				Ferrimonadaceae	3.4%	0.6%	0.6%	1.0%	0	44	33	44
				Idiomarinaceae	1.7%	1.4%	0.3%	0.6%	4	54	33	63
				Moritellaceae	0.0%	1.1%	0.0%	0.0%	22	33	56	44
				Pseudoalteromonadaceae	0.7%	0.0%	0.0%	0.3%	30	36	59	55
				Psychromonadaceae	1.9%	1.5%	0.0%	0.0%	14	43	29	64
				Shewanellaceae	1.1%	1.8%	0.3%	0.4%	18	39	23	46
			Cardiobacteriales	Cardiobacteriaceae	12.4%	5.3%	3.2%	3.1%	0	0	0	0
			Chromatiales	Chromatiaceae	3.6%	2.8%	2.0%	1.6%	0	12	3	17
				Ectothiorhodospiraceae	4.6%	4.2%	3.7%	3.3%	0	0	0	0
				Halothiobacillaceae	10.8%	3.7%	4.2%	3.5%	0	0	0	0
				Thioalkalispiraceae	5.3%	0.9%	0.7%	1.1%	0	0	0	0
			Enterobacteriales	Enterobacteriaceae	2.3%	1.6%	0.3%	0.6%	12	42	24	36
			Legionellales	Coxiellaceae	22.5%	13.8%	15.5%	14.7%	0	0	0	0
				Legionellaceae	3.7%	1.8%	1.0%	1.0%	0	31	22	35
			Lysobacteriales	Lysobacteraceae	2.7%	1.6%	1.3%	1.0%	3	27	27	37
				Nevskiaceae	2.6%	3.5%	2.8%	2.6%	0	0	0	14
				Sinobacteraceae	4.0%	1.2%	0.4%	0.3%	0	50	33	50
			Methylococcales	Methylococcaceae	2.1%	2.2%	2.0%	2.0%	14	17	14	11
			Oceanospirillales	Alcanivoracaceae	2.3%	1.4%	0.7%	0.4%	13	63	38	50
				Hahellaceae	5.9%	2.0%	1.7%	1.8%	0	23	15	31
				Halomonadaceae	2.7%	1.4%	0.7%	0.4%	5	39	30	45
				Litoricolaceae	4.3%	2.8%	1.9%	3.8%	0	0	0	0
				Oceanospirillaceae	4.6%	2.8%	1.0%	0.9%	0	21	5	34
				Saccharospirillaceae	4.3%	2.2%	1.0%	1.0%	0	33	33	33
			Orbales	Orbaceae	6.6%	3.1%	1.2%	3.9%	0	0	0	0
			Pasteurellales	Pasteurellaceae	5.3%	3.0%	1.3%	1.4%	0	26	0	32
			Pseudomonadales	Moraxellaceae	2.6%	1.6%	0.7%	0.8%	5	40	27	33
				Pseudomonadaceae	0.7%	1.2%	0.3%	0.6%	32	34	22	36
			Salinisphaerales	Salinisphaeraceae	3.1%	1.0%	0.8%	1.2%	0	43	43	14
			Thiotrichales	Francisellaceae	0.3%	0.2%	0.0%	0.2%	44	44	56	67
				Piscirickettsiaceae	4.1%	2.0%	1.0%	0.8%	0	34	17	38
				Thiotrichaceae	2.1%	0.9%	1.2%	1.2%	8	0	0	0
			Vibrionales	Vibrionaceae	1.8%	1.6%	0.7%	0.4%	18	40	28	50
	Spirochaetes	Spirochaetes	Spirochaetales	Brachyspiraceae	0.7%	0.2%	0.3%	0.2%	57	57	71	57
				Leptospiraceae	0.7%	0.0%	0.0%	0.0%	39	35	43	39
				Spirochaetaceae	3.0%	1.6%	1.7%	1.4%	0	14	18	9
	Synergistetes	Synergistia	Synergistales	Synergistaceae	3.6%	1.7%	1.7%	2.0%	0	14	10	5
	Tenericutes	Mollicutes	Acholeplasmatales	Acholeplasmataceae	5.7%	3.5%	2.9%	2.5%	0	0	0	0
			Anaeroplasmatales	Anaeroplasmataceae	4.3%	2.9%	2.7%	2.0%	0	0	0	0
			Entomoplasmatales	Entomoplasmataceae	0.6%	0.0%	0.0%	0.0%	31	31	38	38
				Spiroplasmataceae	1.0%	0.8%	1.0%	0.8%	31	34	43	40
			Mycoplasmatales	Mycoplasmataceae	8.0%	3.6%	2.0%	2.4%	0	15	0	8
	Thermodesulfobacteria	Thermodesulfobacteria	Thermodesulfobacteriales	Thermodesulfobacteriaceae	1.8%	2.4%	1.7%	1.7%	13	50	0	38
	Thermotogae	Thermotogae	Kosmotogales	Kosmotogaceae	10.8%	0.2%	0.2%	0.1%	0	0	0	0
			Petrotogales	Petrotogaceae	1.3%	0.4%	0.1%	0.1%	17	67	50	67
			Thermotogales	Fervidobacteriaceae	3.1%	1.7%	2.0%	2.7%	0	0	20	20
				Thermotogaceae	2.8%	1.0%	1.1%	1.2%	4	29	18	25
	Verrucomicrobia	Opitutae	Puniceicoccales	Puniceicoccaceae	4.5%	2.6%	0.8%	1.6%	0	29	0	29
		Spartobacteria	Spartobacteria	Verrucomicrobiaceae	5.0%	3.0%	1.7%	1.6%	0	15	0	27
		Verrucomicrobiae	Verrucomicrobiales	Rubritaleaceae	1.2%	0.6%	0.3%	0.2%	33	50	33	50
Archaea	Crenarchaeota	Thermoprotei	Desulfurococcales	Desulfurococcaceae	15.7%	13.1%	6.0%	7.2%	0	0	0	0
			Sulfolobales	Sulfolobaceae	3.8%	5.9%	5.2%	6.3%	0	0	0	0
			Thermoproteales	Thermoproteaceae	11.2%	1.8%	1.4%	3.0%	0	0	10	0
	Euryarchaeota	Archaeoglobi	Archaeoglobales	Archaeoglobaceae	17.9%	7.9%	4.7%	3.7%	0	0	0	0
		Halobacteria	Halobacteriales	Halobacteriaceae	19.0%	13.1%	8.2%	7.3%	0	0	0	0
		Methanobacteria	Methanobacteriales	Methanobacteriaceae	11.2%	5.2%	3.8%	3.7%	0	0	0	0
		Methanococci	Methanococcales	Methanocaldococcaceae	17.2%	6.7%	2.0%	3.7%	0	13	0	0
				Methanococcaceae	16.8%	22.1%	6.1%	7.4%	0	0	0	0
		Methanomicrobia	Methanocellales	Methanocellaceae	29.6%	9.8%	3.7%	7.1%	0	0	0	0
			Methanomicrobiales	Methanocorpusculaceae	23.7%	1.5%	1.5%	2.1%	0	0	0	0
				Methanomicrobiaceae	4.9%	3.4%	3.3%	3.3%	0	0	0	0
				Methanoregulaceae	14.6%	10.1%	5.8%	5.9%	0	0	0	0
				Methanospirillaceae	30.8%	7.5%	3.6%	2.6%	0	0	0	0
			Methanosarcinales	Methanosarcinaceae	13.1%	3.7%	2.0%	2.0%	0	10	0	14
				Methanotrichaceae	69.5%	34.6%	9.5%	10.1%	0	0	0	0
		Thermococci	Thermococcales	Thermococcaceae	1.2%	0.0%	0.0%	0.2%	14	47	42	56
		Thermoplasmata	Thermoplasmatales	Ferroplasmaceae	0.0%	0.0%	0.0%	0.0%	0	0	0	0
Average					6.5%	3.1%	2.3%	2.3%	7	25	22	28

Supplementary file4: Distances and percentage improvement when applying DynamiC compared to the default cut-off for the nowadays commonly used amplicons of the 16S rRNA gene. The percentage improvement range from red (minimum) to green (maximum), while the distances follow the heatmap legend.

0.0%	0.5%	1.0%	1.5%	2.0%	2.5%	3.0%	>3.0%
