# Supplementary material for: Reconciliation between operational taxonomic units and species boundaries
Source: FEMS Microbiol Ecol. 2017 Mar 21;93(4):fix029. doi: 10.1093/femsec/fix029 (PMC5812548; doi:10.1093/femsec/fix029)
Supplement: Supplemental material — Supplementary data are available at FEMSEC online. [file fix029_supp.zip › Supplementary_file5.pdf]

Supplementary file 5: Expected number of OTUs together with the number of total, over-split, and over-merged OTUs obtained using both the 97% cut-off versus DynamiC for the 34 mock samples.

Meta ID	Expected number of OTUs	Total Number of OTUs	# over-merged OTUs	# over-split OTUs	Total Number of OTUs	# over-merged OTUs	#over-split OTUs
		97% Cut-off			Lookup table		
**35**	51	46	7	2	52	3	4
**36**	47	44	5	2	48	3	4
**37**	50	45	7	2	49	3	2
**38**	45	43	5	3	47	3	5
**42**	46	43	5	2	45	4	3
**43**	46	43	5	2	45	3	2
**44**	46	43	5	2	45	3	2
**45**	46	42	5	1	44	3	1
**46**	46	42	5	1	43	4	1
**47**	44	38	6	0	40	4	0
**48**	40	37	5	2	40	3	3
**49**	41	39	5	3	46	3	8
**59**	48	48	5	5	52	4	8
**60**	49	49	5	5	51	3	5
**61**	48	48	5	5	49	3	4
**66**	43	41	5	3	46	3	6
**74**	44	42	5	3	45	3	4
**75**	46	44	5	3	47	3	4
**76**	45	43	5	3	47	3	5
**77**	43	37	6	0	38	5	0
**80**	46	42	5	1	43	4	1
**81**	45	41	5	1	43	3	1
**85**	46	42	5	1	43	4	1
**86**	47	43	6	2	46	3	2
**87**	47	43	6	2	46	3	2

**88**	49	44	7	2	48	3	2
**89**	46	39	7	0	42	5	1
**90**	46	39	7	0	41	5	0
**91**	46	39	7	0	41	5	0
**98**	43	38	5	0	39	4	0
**99**	43	37	6	0	39	5	1
**100**	43	38	5	0	39	4	0
**101**	43	37	6	0	38	5	0
**102**	43	37	6	0	38	5	0
**Average**	**46**	**42**	**6**	**2**	**44**	**4**	**2**
