# Supplementary figures and images for: Reconciliation between operational taxonomic units and species boundaries
Source: FEMS Microbiol Ecol. 2017 Mar 21;93(4):fix029. doi: 10.1093/femsec/fix029 (PMC5812548; doi:10.1093/femsec/fix029)

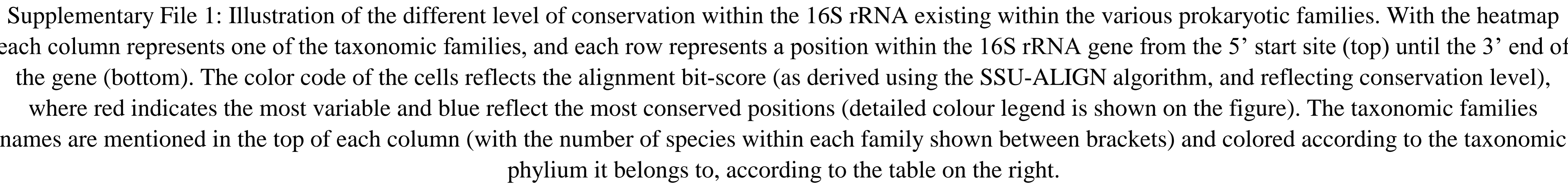

Supplement: Supplemental material — Supplementary data are available at FEMSEC online. [file fix029_supp.zip › Supplementary_file1.pdf]

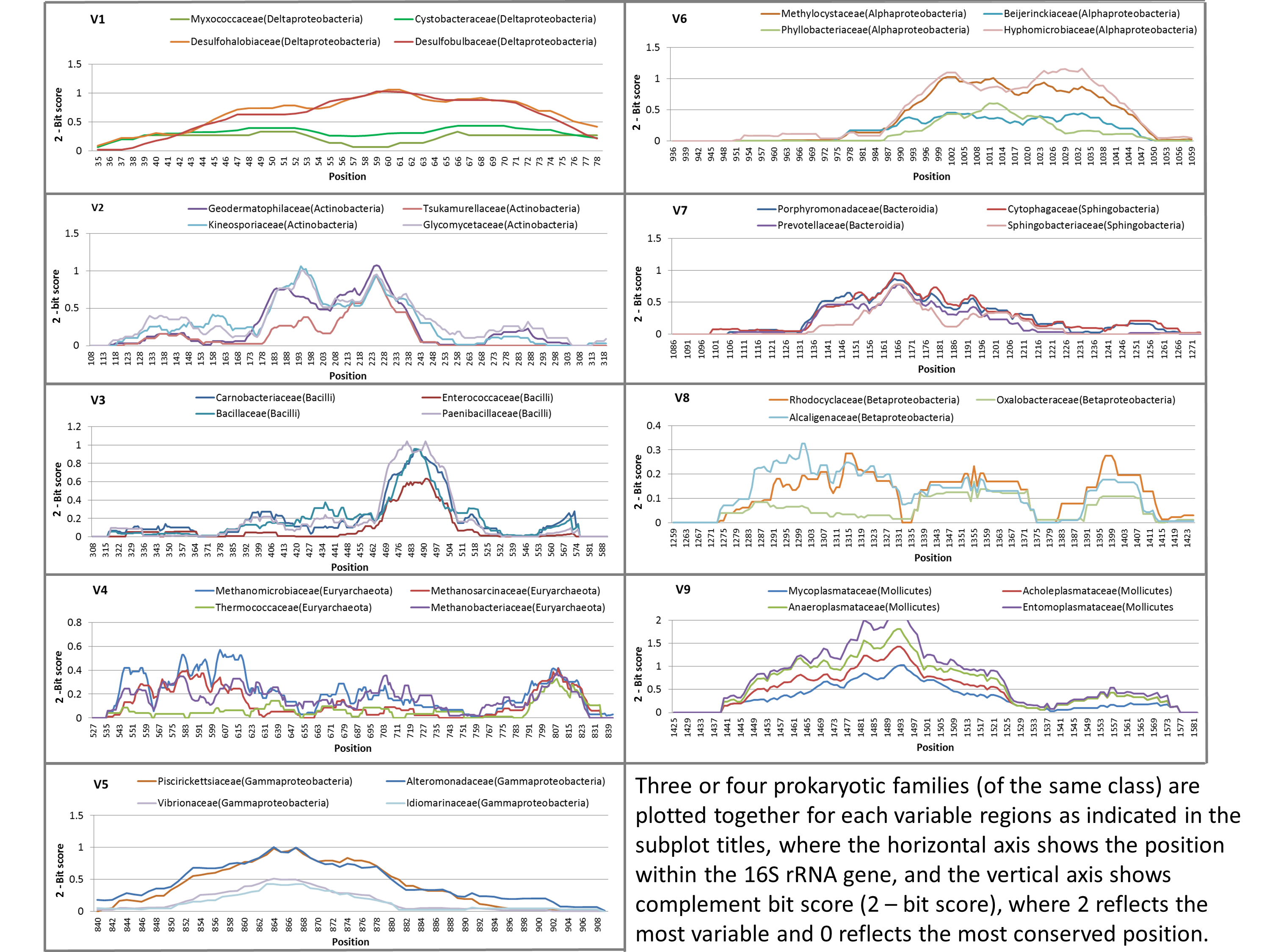

Supplement: Supplemental material — Supplementary data are available at FEMSEC online. [file fix029_supp.zip › Supplementary_file2.tif]

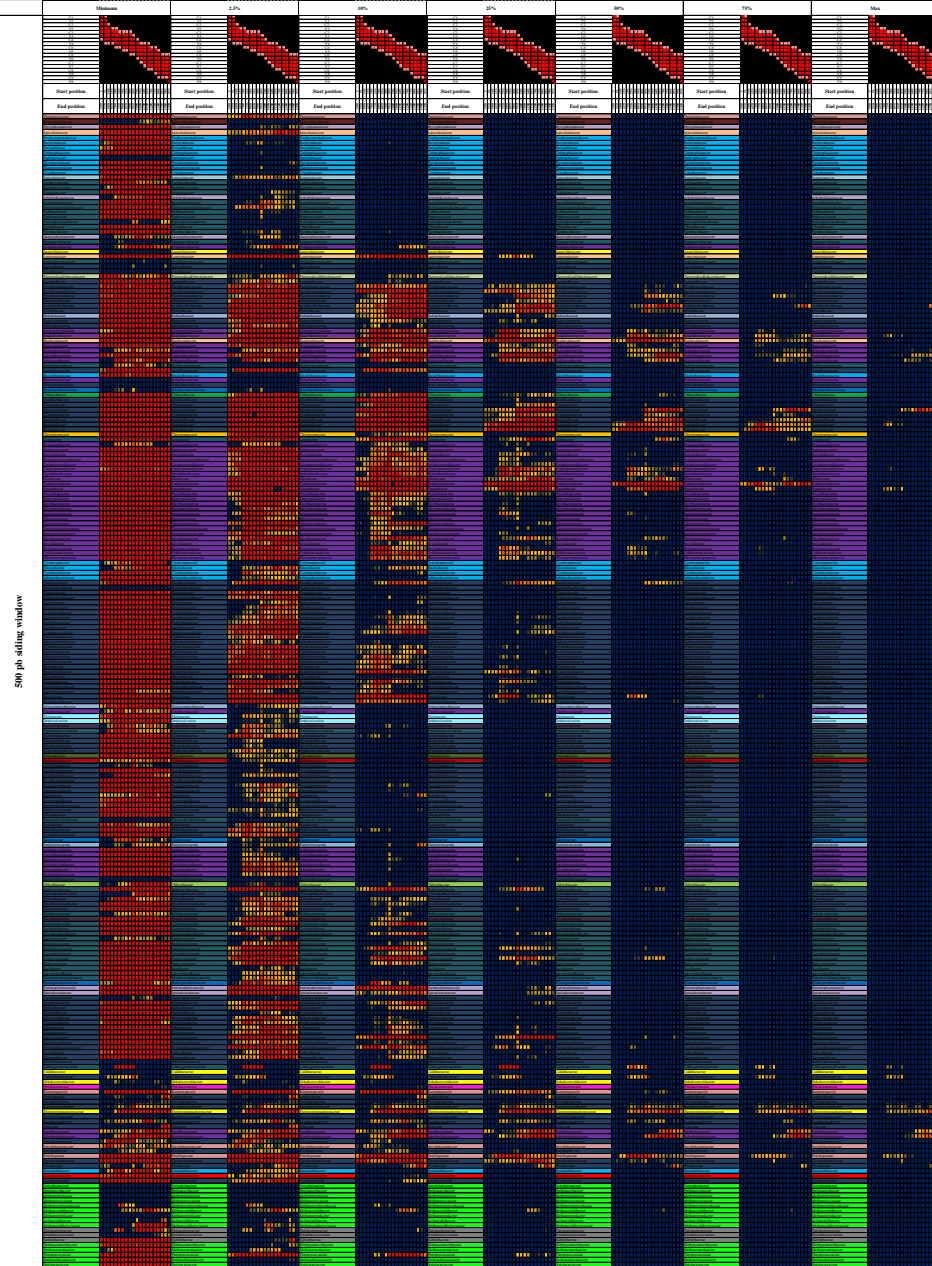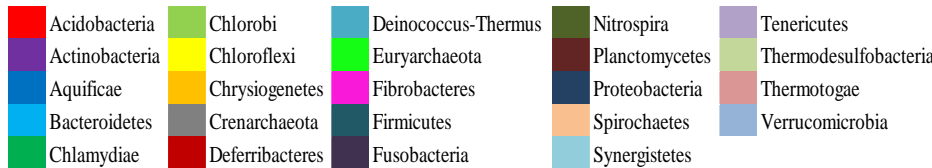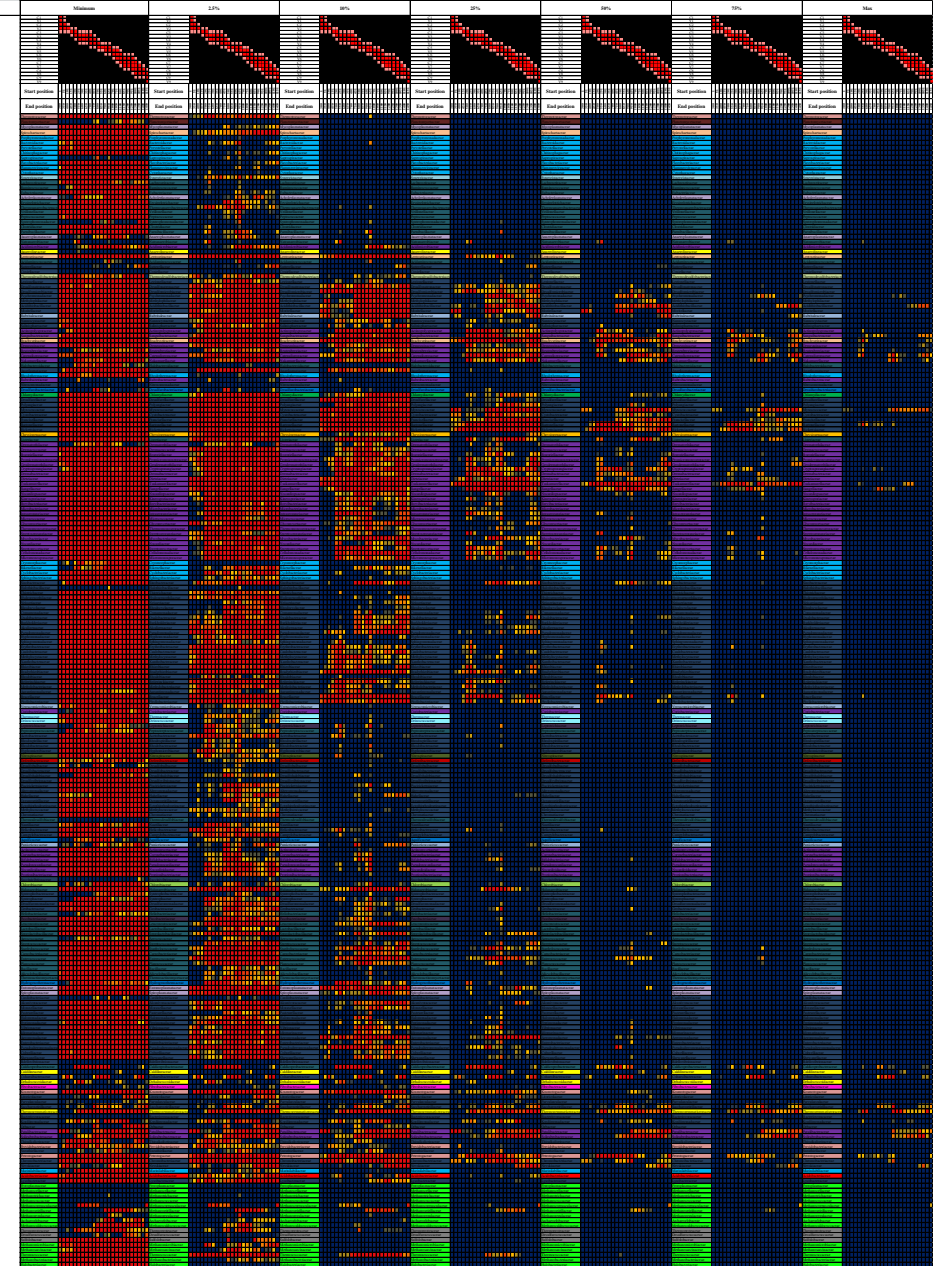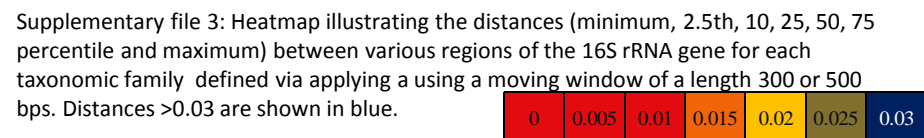

Supplement: Supplemental material — Supplementary data are available at FEMSEC online. [file fix029_supp.zip › Supplementary_file3.pdf]
